# Label-Free Optical Resonator-Based Biosensors

**DOI:** 10.3390/s20205901

**Published:** 2020-10-19

**Authors:** Donggee Rho, Caitlyn Breaux, Seunghyun Kim

**Affiliations:** Electrical and Computer Engineering Department, Baylor University, One Bear Place #97356, Waco, TX 76798, USA; donggee_rho@baylor.edu (D.R.); Caitlyn_Breaux@baylor.edu (C.B.)

**Keywords:** optical resonators, Fabry-Perot interferometer, whispering gallery mode resonators, label-free biosensors

## Abstract

The demand for biosensor technology has grown drastically over the last few decades, mainly in disease diagnosis, drug development, and environmental health and safety. Optical resonator-based biosensors have been widely exploited to achieve highly sensitive, rapid, and label-free detection of biological analytes. The advancements in microfluidic and micro/nanofabrication technologies allow them to be miniaturized and simultaneously detect various analytes in a small sample volume. By virtue of these advantages and advancements, the optical resonator-based biosensor is considered a promising platform not only for general medical diagnostics but also for point-of-care applications. This review aims to provide an overview of recent progresses in label-free optical resonator-based biosensors published mostly over the last 5 years. We categorized them into Fabry-Perot interferometer-based and whispering gallery mode-based biosensors. The principles behind each biosensor are concisely introduced, and recent progresses in configurations, materials, test setup, and light confinement methods are described. Finally, the current challenges and future research topics of the optical resonator-based biosensor are discussed.

## 1. Introduction

Cancers, cardiovascular diseases, and infectious diseases are the leading causes of death worldwide [1,2]. Early and accurate diagnosis of those diseases is vital to allow prompt and effective clinical treatments for patients, which prevents further deterioration and increases the chance of full recovery [3]. Numerous efforts have been invested into advancing medical diagnostic technologies, including many biosensor researches. A biosensor is defined as an analytical device using a biological recognition element in close contact with a transducer [4] to detect and quantify target biological analytes in a sample fluid [5]. The most widely used and commercialized biosensors are enzyme-linked immunosorbent assays (ELISA) and surface plasmon resonance (SPR) [6]. ELISA has been considered as a standard method for identifying and quantifying the target analyte with high sensitivity and specificity, including antigens/antibodies, proteins, and hormones [1,7]. It is a labeled immunoassay, however, involving laborious and time-consuming preparation steps with sophisticated and costly instruments needed to be operated by a trained expert working in a centralized clinical laboratory [7,8,9]. SPR-based biosensors are attractive because of their label-free operation and real-time analysis capability [10,11,12,13,14,15]. They detect biomolecular interactions occurring on the metal surface with high sensitivity by measuring the shift in the excitation angle of surface plasmon or the optical intensity change. Nonetheless, it requires complicated and expensive settings [16] limiting the possibility to miniaturize the system [17,18]. 

Optical resonator-based biosensors are actively investigated to achieve a highly sensitive detection down to single bio-molecule interactions without labeling [19,20,21,22]. Optical resonators are inherently sensitive to the changes in the surrounding environment due to their strong light confinement capability and resonance characteristic [23]. With the recent development in microfluidic platforms and microfabrication technologies, optical resonator-based biosensors are further miniaturized [24] and regarded as a promising point-of-care (POC) device platform for the early and highly accurate detection of diseases. POC devices allow medical diagnostic tests to be conducted at or near the patients, which eventually increases the chance of early detection of diseases and satisfies the need for in-home healthcare and resource-limited settings [25]. To be effectively used in POC diagnostics, the biosensor must have several characteristics such as label-free detection, low-cost, high sensitivity, high selectivity, simultaneous detection capability of multiple analytes with a small sample volume, and portability [25].

In this review, we aim to summarize the recent development of label-free optical resonator-based biosensors. Optical resonator-based biosensors are categorized into two groups, Fabry-Perot Interferometer (FPI)-based biosensors and whispering gallery mode (WGM)-based biosensors, based on the light confinement mechanism [26]. FPI-based biosensors are divided into three sections: (1) FPI with two reflecting surfaces, (2) porous silicon microcavity (PSM), and (3) optical fiber-based FPI. WGM-based biosensors are categorized into (1) ring and disk resonators, (2) spherical resonators, (3) toroid resonators, and (4) cylindrical or capillary-based resonators. For each of them, basic principles, sensing mechanisms, performance, and recent advancements are discussed.

## 2. Optical Resonator-Based Biosensors

An optical resonator is designed to allow light waves at specific (resonant) frequencies to be confined and stored within [17,27,28]. Light waves continuously travel back-and-forth in between two or more interfaces or circulate within the optical resonator structure. At a resonant frequency, light waves constructively interfere without suffering significant losses, while light waves at other frequencies are suppressed with destructive interference. The resonant frequencies are determined by the material and geometry of the optical resonator. They are highly sensitive to changes in the temperature, pressure, and refractive index of the surrounding medium. The sensitivity of an optical resonator-based biosensor is related to the sharpness of the resonance response, which is expressed as the quality factor (Q-factor) of an optical resonator. The Q-factor is defined as

(1) $$Q\;=\;\frac{\;v_{m}}{\Delta v}$$

where *ν_m_* is the resonant frequency and ∆*ν* is the full width at half maximum (FWHM) at the resonant frequency [29]. For a specific resonant frequency, the Q-factor increases as the width of the resonant curve is reduced. 

For an optical resonator to be used as a biosensor, a bio-recognition element or a bioreceptor, which has a specific affinity to an analyte of interest in a biological sample fluid, is functionalized onto it. As the target analytes in a sample fluid attach to the bioreceptor, the effective refractive index of the surrounding medium is changed, resulting in a shift in the resonant spectrum. Either a shift in the resonant wavelength or a change in light intensity is measured with a readout element to quantify the concentration of the analyte in a sample medium and determine the binding kinetics from real-time detection data.

Two measures, sensitivity and limit of detection (LOD), are commonly used to determine the quality of an optical resonator-based biosensor. The sensitivity (S) of an optical resonator-based biosensor is equal to the amount of shift in resonant frequency (or wavelength) per change in refractive index or molecular concentration [30] (equivalent to the slope of a calibration curve [31]) and calculated by

(2) $$S\;=\;\frac{\Delta \lambda }{\Delta n}\;\left[\frac{nm}{RIU}\right]$$

where ∆*λ* is the shift in resonant wavelength and ∆*n* is the change in refractive index. 

The LOD is the minimum detectable concentration of a biosensor. If it is not experimentally determined, the LOD is often estimated. The way to estimate the LOD may differ depending on the signal-to-noise ratio and the governing entity in the respective field of the work [32,33], but, for most cases, the LOD of a biosensor is estimated using the standard deviation (*σ*) by

(3) LOD=3 × σ

while some variations include a coefficient of 3.3 in the place of 3 as suggested by the International Organization for Standardization (ISO), and LCGC Europe [33]. The standard deviation is usually obtained by measuring the signal on a sample with a null concentration or blank [31,34]. 

## 3. Fabry-Perot Interferometer (FPI)-Based Biosensors

The FPI, called an etalon or an optical cavity, has the simplest structure out of many different geometries of the optical resonators, confining the light between two parallel reflecting surfaces. The simple structure of the FPI allows it to be easily combined with a microfluidic system and integrated with other optical materials or components such as a porous silicon or an optical fiber [35,36,37,38]. FPI-based biosensors boast a high sensitivity compared to other interferometric biosensors based on Mach-Zehnder and Michelson interferometers due to its resonance characteristic through a large number of reflections [39,40]. 

**Figure 1:**
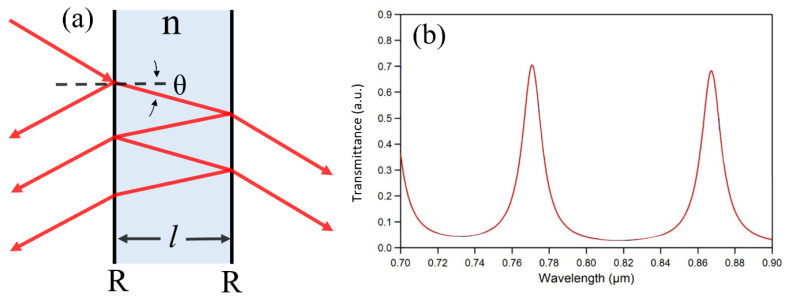
(**a**) Structure of FPI. (**b**) Transmission spectrum of FPI.

The incident light onto the FPI undergoes multiple reflections and transmissions at two reflecting surfaces, creating numerous reflected and transmitted waves, as shown in Figure 1a. 

The transmittance (T) of the optical cavity can be represented as

(4) $$T\;=\;\frac{I_{t}}{I_{i}}\;=\frac{{\left(1\;-\;R\right)}^{2}}{\;{\left(1-R\right)}^{2\;}+\;4{\mathrm{Rsin}}^{2}\left(\;\frac{\delta }{2}\;\right)}$$

where *I_t_* and *I_i_* are the transmitted intensity and incident intensity, respectively, and R is the reflectance of the surfaces, assuming the reflectance of the reflecting surfaces are the same. The phase difference (δ) is given by

(5) $$\delta \;=\;\left(\frac{2\pi }{\lambda }\right)\cdot 2nl\cos\theta$$

where *λ* is the wavelength of light in vacuum, *n* is the refractive index inside the optical cavity, *l* is the distance between reflecting surfaces, and θ is the angle of the light propagating inside the cavity. If the phase difference is equal to an integer multiple of 2π, the transmittance becomes maximized where the constructive interference occurs. However, the transmitted waves will destructively interfere with each other when the phase difference becomes an odd integer multiple of π, leading to the minimized transmittance. Therefore, the transmission spectrum as a function of the wavelength shows a cyclic resonance characteristic with peaks and valleys as represented in Figure 1b. 

Under normal incidence, the resonant frequencies (*ν_m_*) can be calculated by

(6) $$v_{m}\;=\;\frac{c}{2nl}m$$

where *c* is the speed of light in vacuum and *m* is the integer, which represents the resonator-mode order. The corresponding free spectral range (FSR)—the peak-to-peak—separation, is also obtained as

(7) $$\mathrm{FSR}\;=\;v_{m+1}\;-\;v_{m}\;=\;\frac{c}{2nl}$$

A sample fluid usually flows through the optical cavity containing the bioreceptor-functionalized internal surface. When target molecules are attached to the immobilized bioreceptors, the local refractive index, where the binding event happens, and the electric field distributions inside the optical cavity are changed. This, in turn, causes the resonance spectrum to be shifted. Most FPI-based biosensors measure the shift in the resonance peak using a spectrometer or a tunable laser, while some technologies measure the intensities at single or multiple wavelength(s).

### 3.1. FPI-Based Biosensors with Two Reflecting Surfaces

**Figure 2:**
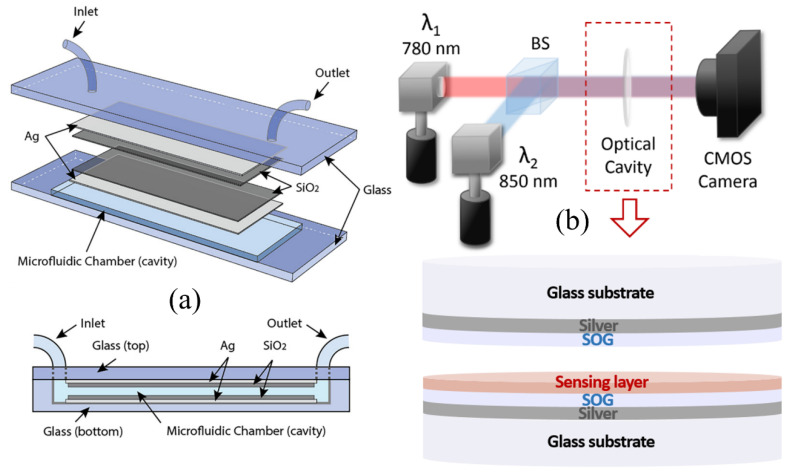
The FPI-based biosensors with two planar-parallel reflecting surfaces. (**a**) Structure of the proposed biosensor in 3D and cross-sectional views. Reproduced from ref. [37] with permission from Elsevier. (**b**) Schematic diagram of the proposed device with the optical cavity structure. Reproduced from ref. [41] with permission © The Optical Society of America.

There have been efforts to develop optical biosensors utilizing the simple structure of FPI to have characteristics such as an easy fabrication process, simple test setup, and cost-effectiveness [35,36,37,38,39,40,41,42,43,44,45,46,47,48,49,50]. For example, You et al. developed a microfluidic enhanced FPI-based biosensor to detect glucose, sodium chloride (NaCl), and potassium chloride (KCl) for diabetes patients [37]. The reflecting surfaces are fabricated by depositing silver (Ag) and silicon dioxide (SiO_2_) thin films as shown in Figure 2a. The different concentrations of glucose, NaCl, and KCl from 5% to 25% were measured by detecting the peak shift using a spectrophotometer. The resolution for the bulk refractive index detection of the proposed device was 10^−5^ refractive index unit (RIU), while the minimum detectable concentrations were determined to be 0.01% glucose, 0.00769% KCl, and 0.00555% NaCl solutions. Our research group developed a low-cost and label-free optical cavity-based (OCB) biosensor for point-of-care (POC) diagnostics [41]. A schematic of the proposed device is shown in Figure 2b. Instead of measuring a shift in the resonant peak using an expensive spectrometer or a costly tunable laser, the proposed biosensor measures the intensities at two specific wavelengths using low-cost laser diodes and a CMOS camera. A differential calculation is employed for the OCB biosensor to enhance the sensitivity, equalize the measured intensity variations for each test, and improve fabrication tolerance [51]. In addition to being low-cost and highly sensitive, the intensity-based detection method enables label-free and multiplexed assays. The OCB biosensor was experimentally demonstrated using standard refractive index fluids to detect the change in bulk refractive index inside the optical cavity, validating benefits of the differential detection method [42,51]. The label-free and real-time biosensing capability of the OCB was demonstrated by detecting biotinylated bovine serum albumin (BSA) with streptavidin as a bioreceptor [41]. The estimated LOD was 2.82 pM, and the potential for multiplexed detection was demonstrated. A portable OCB is prototyped with 3D printed parts and off-the-shelf optical components at a cost of less than $1500 [38]. The LOD of the portable system was determined to be 1.73 × 10^−5^ RIU from refractive index measurements.

**Figure 3:**
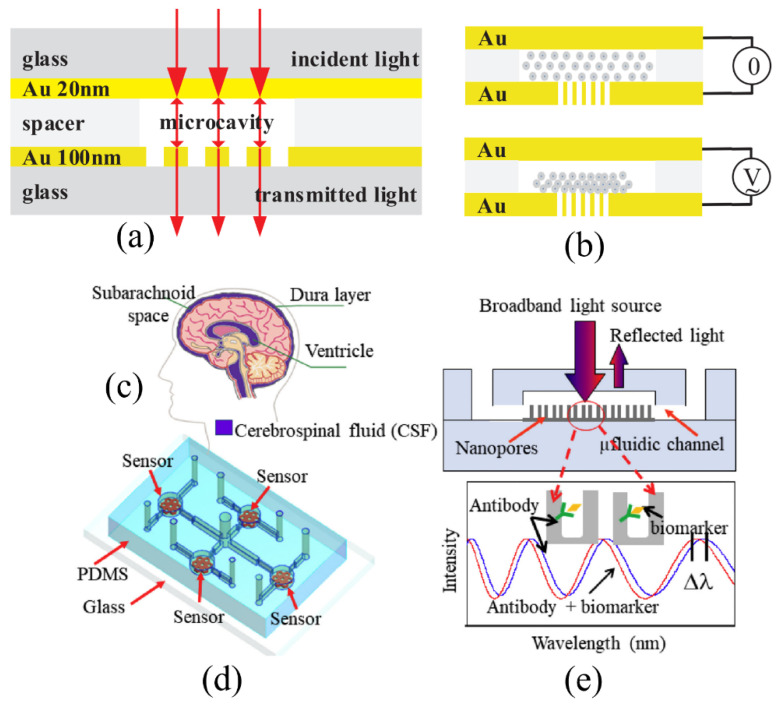
Nanostructures employed at one side of the FPI. (**a**) Schematic of the μFPI. (**b**) Concept of DEP. Reproduced from ref. [43] with permission from Elsevier. (**c**) The indicated location of CSF. (**d**) Schematic of a chip with four nanoFPIs. (**e**) The working principle of nanoFPI. Reproduced from ref. [45] with permission from Elsevier.

Some researchers have tried to modify the FPI structure to further enhance the sensitivity of the FPI-based biosensor. For example, Tu et al. reported a micromachined-FPI (μFPI) with a perforated gold layer with nanohole arrays on a reflecting surface to increase the sensing surface area as shown in Figure 3a [43]. The nanohole arrays have a diameter of 200 nm and a period of 500 nm fabricated using focused ion beam (FIB) milling. The sensitivity obtained from refractive index detection was determined to be 593 nm/RIU with a Q-factor of 128.4. The dielectrophoresis (DEP) technique was employed to concentrate molecules near the nanoholes using an external electric field, as illustrated in Figure 3b. With this technique, the concentration of BSA was measured down to 1 pM. In a similar work, Zhang et al. developed a nanostructured-FPI (nanoFPI) device with an embedded nanopore layer inside the cavity [44]. The Au-coated nanopore layer with a size of 50 nm is created to increase the sensing area and enhance the sensitivity as shown in Figure 3e. The same research group employed the nanoFPI to detect beta-amyloid (Ab42) and total tau (T-tau) biomarkers in cerebrospinal fluid (CSF) for the diagnosis of Alzheimer’s diseases [45]. One sensor chip has four nanoFPIs as shown in Figure 3d, and each of them has its own sub-inlet and sub-outlet to functionalize two different antibodies, allowing the multiplexed detection of Ab42 and T-tau. They were able to detect Ab42 and T-tau in buffer down to 7.8 pg/mL and 15.6 pg/mL, respectively, validating the specificity by introducing Ab42-buffer to the T-tau antibody-functionalized sensor and vice versa. The CSF samples spiked with the Ab42 concentrations from 31.25 pg/mL to 500 pg/mL and the T-tau concentrations from 125 pg/mL to 2000 pg/mL were measured showing the potential to monitor multiple biomarkers in clinical samples.

**Figure 4:**
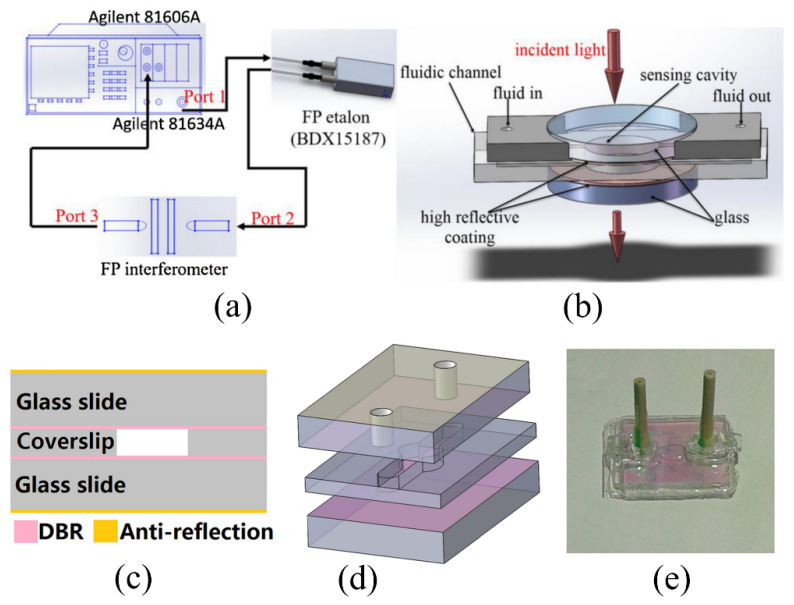
(**a**) Schematic of the two cascaded FPIs system. (**b**) The sensing FPI. Reproduced from ref. [52] with permission from Elsevier. (**c**) Schematic of the FPI employing two DBRs. (**d**) Fabrication process. (**e**) Image of the fabricated sensor. Reproduced from ref. [36] with permission © The Optical Society of America.

A cascaded FPI sensor was also proposed to achieve the improved sensitivity of the FPI-based biosensor [52]. It consists of a tunable laser source, a reference cavity, an optical fiber between cavities, a sensing cavity, and a power sensor as shown in Figure 4a,b. The transmission spectrum of the cascaded FPIs shows a higher Q-factor and a larger FSR due to the Vernier effect. The different concentrations of NaCl were measured in triplicate, and the sensitivity of bulk refractive index change was obtained as 23,794.6 nm/RIU for the resonant wavelength shift, which corresponds to the LOD of 8.4 × 10^−7^ RIU. For the intensity-based measurement at a fixed wavelength, the LOD was obtained to be around 1.3 × 10^−5^ RIU. Different concentrations of Streptomycin-BSA conjugate antigen in the range of 1–7 ng/mL were detected with the cascaded FPI biosensor. For the experiments, Streptomycin-BSA is mixed with Streptomycin monoclonal antibody and introduced into the channel. The refractive index difference due to the different concentration of Streptomycin-BSA produces measurable signal changes. The specificity of the device was also validated by introducing Kanamycin and phosphate buffer saline (PBS) to confirm negligible wavelength shifts from their interactions with a Streptomycin monoclonal antibody.

Other researchers employed distributed Bragg reflectors (DBRs) as reflecting surfaces for the FPI to achieve a high Q-factor [35,36,46]. The DBR consists of two alternating dielectric layers with high and low refractive indices, acting as a highly reflecting region with a low absorption loss. For instance, Wu et al. reported an all-glass FPI-based biosensor using DBRs as partially reflecting regions as shown in Figure 4c–e [36]. The DBR consisted of 3.5 pairs of SiO_2_ and tantalum pentoxide (Ta_2_O_5_) deposited on a glass slide. Two of the slides were separated by a coverslip and bonded using UV adhesive. The device was able to detect 15 ng/mL of glucose solution, which corresponds to 2 × 10^−9^ RIU, with a Q-factor of 875.

**Figure 5:**
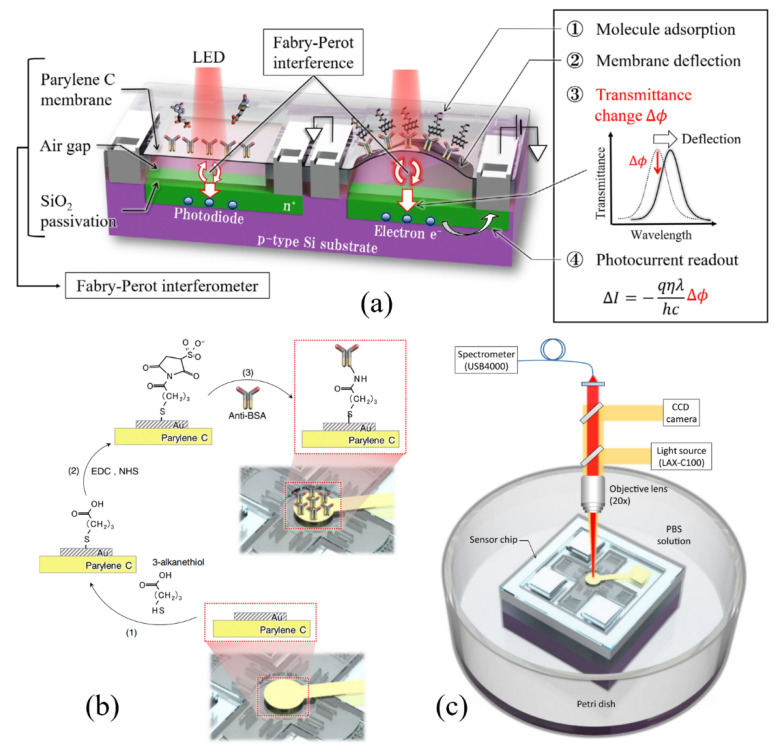
Schematics of the FPI-incorporated surface stress sensor. (**a**) Transducing procedure showing the deflection of the movable membrane film as target molecules adsorb to it. (**b**) Anti-BSA immobilization. (**c**) Spectrum measurement. Reproduced from ref. [40] with permission from IOPscience.

The simplicity of the FPI structure is ideal to develop various configurations by incorporating other structures with it. For example, Takahashi et al. utilized an FPI to improve the sensitivity of a microelectrochemical system (MEMS)-based surface stress sensor for label-free biosensing [40]. The FPI structure consists of two gold (Au) layers with a thickness of 50 nm for highly reflecting surfaces. The top Au layer is deposited at the center of the parylene C film, which acts as a movable membrane as shown in Figure 5a. To demonstrate the label-free biosensing, the Au film on the movable membrane was functionalized with BSA antibodies as seen in Figure 5b. As BSA molecules in PBS buffer solution are introduced and captured by the antibodies, the movable membrane is deflected by the surface stress change, which leads to a displacement in the optical cavity length and a shift in the reflection spectrum measured by a spectrometer in the test setup (Figure 5c). The proposed FPI-based biosensor was able to detect 10 ng/mL of BSA. To evaluate the response from nonspecific binding, 100 ng/mL of avidin was introduced showing 2.4 times smaller deflection than that measured with 10 ng/mL of BSA.

**Figure 6:**
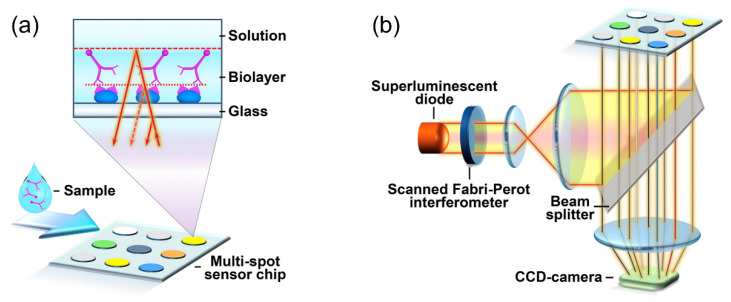
(**a**) Schematic of the biolayer created on the microarray biochip. (**b**) Schematic of the optical system for the proposed biosensor. Reproduced from ref. [53] with permission from Elsevier.

Orlov et al. reported a biosensor employing the spectral correlation interferometry (SCI) using two FPI interferometers and a microarray glass biochip for high sensitivity and simultaneous detection of multiple target analytes [53,54]. As shown in Figure 6a, multiple antigens are immobilized on a microarray biochip at different spots to detect target autoantibodies. The increase in the biolayer thickness with the binding of autoantibodies is measured by the phase change of the correlation signal of two interferometers, piezoelectric actuated FPI and the interferometer created by the glass surface and the interface between the biolayer and sample fluid. For the simultaneous detection in multiple spots, the image signals obtained by a CCD camera are averaged over the area of each spot as shown in Figure 6b. Anti-thyroglobulin (anti-TG) and anti-thyroid peroxidase (anti-TPO) for the diagnosis of autoimmune thyroid diseases were detected in human blood serum with LODs of 1.7 IU/mL and 6 IU/mL, respectively, and their native kinetics in 25 min. 

### 3.2. Porous Silicon Microcavity (PSM)-Based Biosensors

A porous silicon microcavity (PSM) is an FPI structure created by two DBRs with a thin layer of porous silicon (PS). PS is inexpensive, easy to fabricate, and has a large sensing surface, so it has been widely investigated to develop label-free optical biosensors [55,56,57,58,59,60,61,62,63,64,65]. The refractive index of a PS layer is determined by the porosity, which can be modulated by adjusting parameters of electrochemical etching such as the etching time and current density. A high porosity produces low refractive index of PS layer and vice versa. A high surface-to-volume ratio can be obtained by a high porosity, which improves the adsorption of target molecules to the immobilized bioreceptors on the internal pore walls. However, there is a tradeoff between the porosity and the durability of the structure. For biosensing tests, as target molecules in a sample fluid are bound inside the pores, the effective refractive index inside the pores is increased, causing a shift in the resonance spectrum.

**Figure 7:**
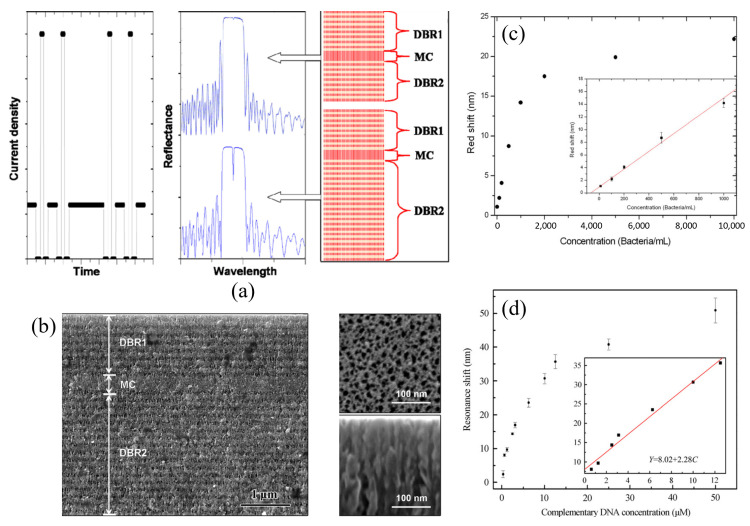
(**a**) (**left**) Current density used to fabricate two DBRs. (**middle**–**right**) Narrow resonance peak using the PSM structure of 8 layers for DBR1 and 20 layers for DBR2. (**b**) SEM images of the PSM. (**c**) PSM measurement results for bacteria concentrations. Reproduced from ref. [58] with permission from IOPscience. (**d**) PSM measurement results for DNA concentrations. Reproduced from ref. [55] with permission from Elsevier.

Li et al. developed a PSM-based biosensor for bacterial detection [58]. The DBR layers were optimized to have 8 periods for the top DBR and 20 periods for the bottom DBR, showing a narrow resonance peak at around 600 nm with an FWHM of 8 nm as shown in Figure 7a. The pore diameter of the PSM structure was measured to be in the range between 5 and 20 nm, and the quality of the pore was evaluated by checking if any blockage appeared on a cross-sectional image as shown in Figure 7b. The surface was modified using undecylenic acid and vancomycin receptor molecules, which have an affinity with various types of bacteria. Because the dimension of bacteria is about 2 μm × 1 μm, which is too big to be infiltrated into the PS, the bacteria wall debris molecules with a diameter of 8 nm were generated by pretreatments. Different concentrations of solutions from 10 to 10,000 bacteria/mL were tested, showing an LOD of 20 bacteria/mL as described in Figure 7c. Other PSM-based biosensors for DNA detection were also reported [55,57]. Zhang et al. employed a PSM-based DNA biosensor using a silicon-on-insulator (SOI) wafer [55]. SOI wafer has benefits in terms of realizing a miniaturized biosensor integrated on a photonic integrated circuit. The PSM on SOI had the average pores diameter of 20 nm to detect 19-base pair DNA with a length of about 4 nm. The reflectance spectrum of the device showed a resonance peak at 1555 nm, with an FWHM of 26 nm. The 3-aminopropyl-triethoxysilane (APTES) and glutaraldehyde are used to immobilize probe DNAs on the PSM. Different concentrations of complementary DNA were measured, ranging between 0.625 and 12.5 μM with an LOD of 43.9 nM as shown in Figure 7d.

**Figure 8:**
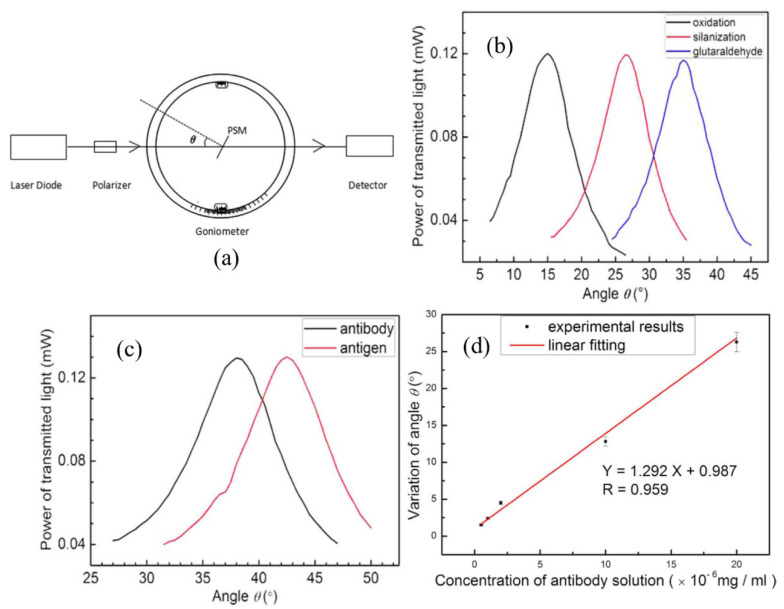
(**a**) Schematic of the spectrometer-free PSM method. (**b**) Shift in transmission angular spectrum during the surface functionalization. (**c**) Shift by the antigen-antibody. (**d**) Measurement results for the concentrations of the hydatid antigen. Reproduced from Open Access Article [61] in Springer Nature.

Another research group developed a spectrometer-free PSM-based biosensor [57]. The PSM was fabricated to have a diameter of pores ranging from 10 to 20 nm for the detection of 8-base pair DNA with a length of about 1.76 nm. Instead of measuring the spectrum using a spectrometer, the proposed system employed a goniometer to change the incident angle of the light to the PSM-based biosensor from a single-wavelength laser source as shown in Figure 8a. As the effective refractive index changed in the PSM, the incident angle at the resonance peak is shifted, which can be translated into the shift in the spectrum. The incident angle was measured after each step of the surface preparation, confirming the sensing capability of the device and the immobilization of the probe DNA on the pores surface. The different concentrations of the complimentary DNA were measured from 0.3125 to 10 μM, and the LOD of the device was calculated to be 87 nM, with an angle resolution of 0.2°. The LOD of refractive index change was 5 × 10^−4^ RIU. The same research group reported another type of spectrometer-free PSM-based biosensor using a transmission angular spectrum [61]. The visible light source was replaced with the near-infrared laser light to reduce the absorption on the PSM and possible damage to biological samples. They demonstrated the transmission angular spectrum-based approach through the detection of hydatid disease antigens. The shift in the spectrum was confirmed by comparing angle measurements obtained from each surface functionalization process and the antigen–antibody interaction as shown in Figure 8b and c. The different concentrations of hydatid antigen from 0.5 to 20 ng/mL in 20 mL solution were infiltrated to the PSM, and the LOD is determined to be 0.16 ng/mL, as shown in Figure 8d. The specificity of the device with the hydatid antibody was demonstrated by observing negligible angle changes from the introduction of the non-hydatid antigen.

**Figure 9:**
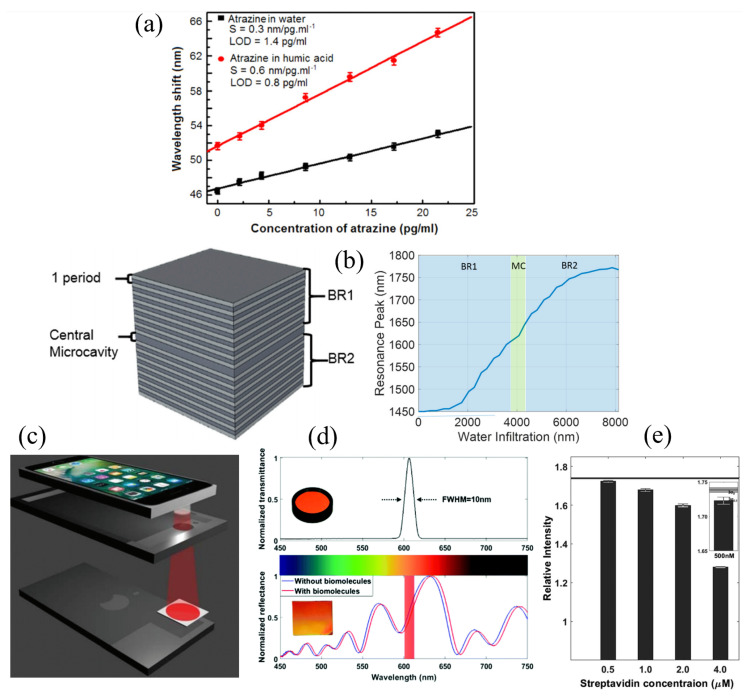
(**a**) Detection of the atrazine pesticide in two different buffers. Reproduced from Open Access Article [59] in IOP Publishing. (**b**) Measured resonance peak in real-time as water infiltrated to PSM. Reproduced from Open Access Article [56] in MDPI. (**c**) Schematic of the smartphone-based system. (**d**) BPF spectrum and the change in normalized transmittance with the adsorption. (**e**) Measurement results for the concentration of streptavidin. Reproduced from ref. [60] with permission from The Royal Society of Chemistry.

Some researchers investigated the potential of PSM-based biosensors for multiplexed, real-time detection, and POC diagnostics [56,59,60]. Pham et al. presented a PSM sensor to detect two different organic solvents simultaneously and measure the low concentration of pesticides in water [59]. As shown in Figure 9a, the atrazine pesticide was detected at different concentrations from 2.15 to 21.5 pg/mL with LODs of 1.4 pg/mL and 0.8 pg/mL in water and humic acid, respectively. The performance of the real-time and in-flow sensing using a PSM-based sensor was evaluated [56]. A fluidic cell incorporated with the PSM was built using poly(methyl methacrylate) (PMMA) for the purpose of enabling the real-time monitoring of the reflectance spectrum as a sample fluid infiltrated the PSM, as described in Figure 9b. The refractive index sensing was done by measuring different concentrations of ethanol in DI water with an LOD of 1000 nm/RIU, corresponding to 10^−7^ RIU. Recently, Cao et al. reported a smartphone-based PSM-biosensor for POC diagnostics [60]. The built-in LED with a band-pass filter (BPF) and the camera of a smartphone were used as a light source and a detector of the platform, respectively, as shown in Figure 9c. A shift in the reflectance spectrum from the adsorption of target molecules on the PSM was detected as an intensity change measured by a smartphone camera. To maximize the relative intensity change from the effective refractive index change, the center wavelength of a BPF was selected to 606.5 nm, which is at between the resonance dip of PSM at near 600 nm and the highest reflectance at near 630 nm as represented in Figure 9d. A customized enclosure was designed to facilitate the alignment of a smartphone with a BPF and a PSM-based biosensor and built with a 3D-printer. The resolution of the intensity change was obtained to correspond with a wavelength shift of 0.33 nm, and the sensitivity of the biosensor for the refractive index change was measured as 350 nm/RIU using glucose solutions. The biosensing capability of the system was demonstrated by measuring different concentrations of streptavidin, and the LOD was estimated to be nearly 500 nM as shown in Figure 9e.

### 3.3. Optical Fiber FPI-Based Biosensors

An optical fiber was originally developed to guide light waves along the core, which is surrounded by a cladding layer through the total internal reflection at the interface with negligible loss. Over the last few decades, optical fibers have been extensively investigated for biosensing applications, with the intrinsic advantages including flexibility, low-cost, small size, and biocompatibility [39,66,67,68,69,70,71,72,73,74,75]. To be developed as a biosensor, the structure of optical fiber has modified to maximize the interaction of light with target analytes and increase the sensitivity [66]. Out of various types of optical fiber-based biosensors, optical fiber FPI-based biosensors have been considered as a promising sensing platform [68,69,76,77,78]. The FPI in the optical fiber is created by splicing it with a different type of optical fiber or depositing a thin reflective layer on the tip. As the effective refractive index or the optical path length between two reflectors of the optical fiber changes, the resonance peak is shifted and measured to determine the concentration of target analytes. 

**Figure 10:**
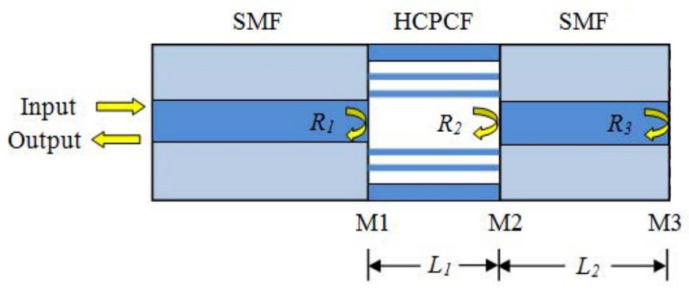
Schematic of the optical fiber FPI. Reproduced from ref. [70] with permission from Elsevier.

Liu et al. reported an inline optical fiber FPI-based biosensor for a label-free immunoassay [69]. The FPI structure was fabricated by cleaving both ends of a hollow-core photonic crystal fiber (HCPCF) and splicing to two single mode fibers (SMFs), defining three reflectors, as shown in Figure 10. The refractive index sensitivity of the sensor was obtained to be 7 × 10^−5^ RIU [70]. To evaluate the proposed device as a label-free immunosensor, the end tip of the SMF was functionalized with goat anti-rabbit IgG using the APTES salinization and glutaraldehyde solution. The concentration of rabbit IgG of 100 ng/mL in PBS was detected in real-time.

**Figure 11:**
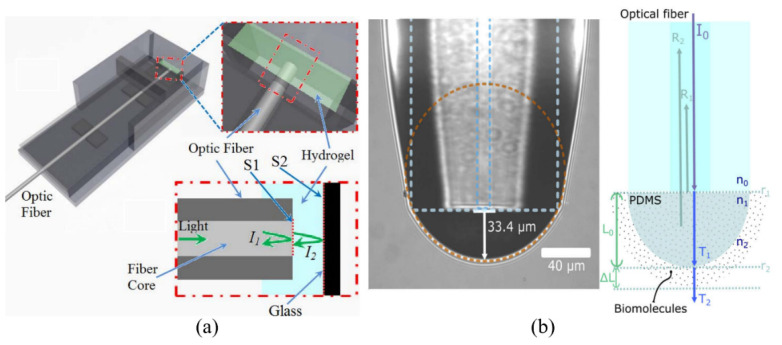
(**a**) Schematic of optical fiber FPI-based sensor using hydrogel. Reproduced from ref. [71] with permission from IEEE. (**b**) Schematic of optical fiber FPI-based sensor using PDMS. Reproduced from ref. [72] with permission © The Optical Society of America.

Without the need of cleaving or splicing, Wu et al. introduced an optical fiber FPI-based biosensor by using a hydrogel to detect bio-toxins [71]. As shown in Figure 11a, two reflective interfaces of this optical fiber FPI-based biosensor are created by the hydrogel layer with a thickness of around 100 μm in between an optical fiber and a glass. The hydrogel was initially fabricated to be water-insoluble, with a crosslinker chosen to be cleaved by a specific agent. The target bio-toxin molecules react with the crosslinker, making it water-soluble. The hydrogel is degraded causing changes in its optical properties. Dithiothreitol (DTT) solutions with different concentrations were applied to the hydrogel, and the resonant spectrum change induced by the degradation of the hydrogel was monitored in real-time. They were able to detect DTT down to 50 μM. Cano-Velazquez et al. also reported a polydimethylsiloxane (PDMS)-coated optical fiber FPI as shown in Figure 11b [72]. The PDMS has been widely used to fabricate optical and microfluidic devices by virtue of the transparency, ease-of-use, and biocompatibility. The hydrophobic PDMS surface was functionalized with mycobacterial lipid antigens, used for tuberculosis diagnosis. The functionalized optical fiber tip was then immersed into Pre-immune serum (PS) and Hyper-immune serum (HS), and the shifts in the resonance peak due to the binding of mycobacterial lipid antigens to anti-lipid antibodies in PS and HS serums were measured in real-time. PS was prepared before immunization having no antibodies, and HS was obtained after immunization with high concentration of antibodies. After compensating the thermal expansion of PDMS, the bio-layer thicknesses created for PS and HS serums were estimated to be 10.78 nm and 30.61 nm, respectively. The antigen coating on the PDMS tip was confirmed to be preserved after exposing it to protein denaturation washes. The results show the potential to realize label-free and real-time detections using a reusable optical immunosensor.

**Figure 12:**
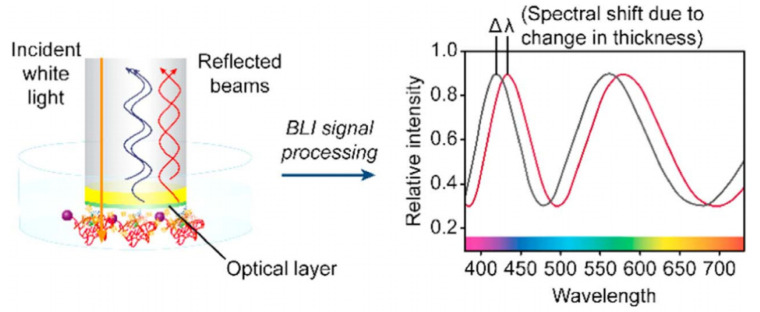
Schematic of BLI. Reproduced from ref. [90] with permission from Elsevier.

Recently, biolayer interferometry (BLI) has been considered as one of the most promising techniques for optical fiber-based, label-free, and real-time detection of biomolecular interactions [79,80,81,82,83,84,85,86,87,88,89,90,91,92,93]. BLI includes two reflective surfaces forming an FPI created by a reference layer at an optical fiber tip and the surface of immobilized bioreceptors as shown in Figure 12 [90]. When white light is propagating toward the fiber tip, the light beams reflected at each of the two surfaces interfere with each other, producing a resonance response in the reflectance spectrum. As the tip of BLI with bioreceptors is dipped into a sample fluid, the target biomolecules bind to the surface, which increases the thickness of the layer and the effective optical length of the FPI, causing the shift in the spectrum. A detector measures the reflectance spectrum in real-time, enabling binding kinetics analyses. BLI has been employed in a wide range of biosensing applications from the study and development of drugs and vaccines [81,83] to the detection of various biomolecules [85,86,87,89,91,92,93,94].

**Figure 13:**
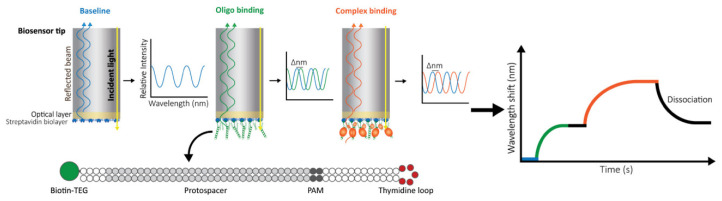
Real-time detection using the tip immobilization procedure. Reproduced from Open Access Article [89] in Frontiers © 2020 Müller-Esparza, Osorio-Valeriano, Steube, Thanbichler and Randau.

For example, Ziu et al. employed the BLI to detect tau441 protein, which is one of the biomarkers of neurodegenerative diseases [80]. One of the commercially available BLI-based biosensors, BLItz instrument (ForteBio, Fremont, CA, USA), was used for the experiments. The tip was functionalized with streptavidin molecules and biotin aptamers. The concentrations of tau441 protein prepared in MES buffer was measured from 0 to 64 nM, with an LOD of 6.7 nM. The selectivity of the sensor was evaluated by confirming negligible signals obtained from other proteins, including amyloid-b_40_, a-synuclein, and BSA. The detection of tau441 in a complex sample, fetal bovine serum (FBS), was conducted using a concentration of 21 nM. The result showed that the wavelength shift was similar to the shift obtained from the result using 21 nM of tau441 in MES buffer. Another work employed the BLItz platform to analyze the interactions between proteins and nucleic acids to characterize CRISPR-Cas systems [89]. The BLI sensor tip is functionalized with streptavidin and biotinylated oligonucleotides. Figure 13 shows the real-time detection results of ribonucleoprotein (RNP) complexes. The complex consists of a CRISPR RNA (crRNA) and CRISPR-associated (Cas) proteins and interacts with a specific anti-CRISPR (Acr) protein. The detection of Acr protein for two different complexes, the Type I-Fv and the Type I-F, was performed with a concentration of 500 nM.

**Figure 14:**
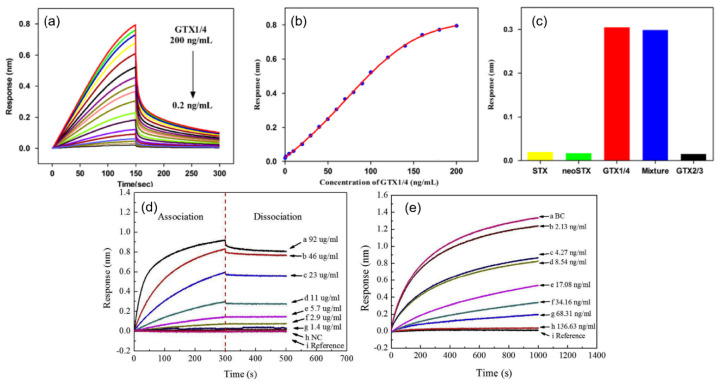
(**a**) Real-time detection after introduction of GTX1/4 at concentrations in between 0.2 and 200 ng/mL. (**b**) Response versus concentrations. (**c**) Evaluation on specificity of the sensor using other toxins. Reproduced from ref. [92] with permission from Elsevier. (**d**) Binding of RepA protein to the dsDNA OP1. (**e**) Measurement results of testosterone. Reproduced from ref. [93] with permission from Elsevier.

The Octet platform (ForteBio, Fremont, CA, USA) is an another commercial BLI-based biosensor, which is capable of detecting biomolecules with a molecular weight of 150 Da, using up to 384-wells for high throughput [79,82,86,95]. The LOD given by the manufacturer falls in the range between nM and μM [86]. Gao et al. reported the detection of Gonyautoxin 1/4 (GTX1/4) aptamer with an Octet platform [92]. It is one of the most well-known neurotoxins to cause paralytic shellfish poisoning (PSP). The GO18-T-d aptamer was used as the bioreceptor, and the concentrations of GTX1/4 were measured in the range from 0.2 ng/mL to 200 ng/mL as shown in Figure 14a,b. The LOD was determined to be 50 pg/mL, and the selectivity of the sensor was evaluated by confirming the negligible cross-reactivity with other toxins, saxitoxin (STX), neoSTX, and GTX2/3 as shown in Figure 14c. The same author also performed the detection of STX with the M-30f anti-STX aptamer using an Octet platform [91]. The different concentrations of STX were measured with an LOD of 0.5 ng/mL. The reusability of the sensor was also evaluated by verifying the remaining sensing capability through four repeated tests.

Another work employing an Octet platform was proposed to quantitatively detect testosterone levels [93]. Biotinylated double-stranded DNA (dsDNA) fragments of operator 1 (OP1) and OP2 were immobilized to the streptavidin-coated surface. Repressor (RepA) proteins attach to the dsDNA OP1, resulting in a resonant wavelength shift for different concentrations as shown in Figure 14d. The presence of testosterone causes a structural change of the RepA protein, leading to the decrease in its binding to dsDNA. Figure 14e shows the real-time measurement results of different concentrations of testosterone down to 2.13 ng/mL, showing the negative correlation with the signal. 

**Figure 15:**
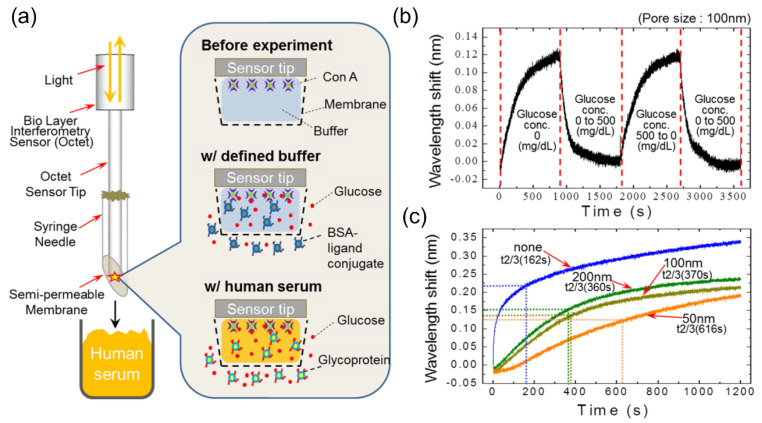
(**a**) Schematic of the needle-type BLI sensor. (**b**) Real-time detection results with a glucose concentration of 500 mg/dL. (**c**) Responses for the different sizes of membrane pore. Reproduced from Open Access Article [96] in MDPI.

Seo et al. developed a needle-type BLI sensor to continuously monitor glucose levels without the use of an enzyme [96]. The needle-type BLI was fabricated by combining a syringe needle with an Octet platform purchased from ForteBio as shown in Figure 15a. The BLI sensor tip was immobilized with concanavalin A (Con A) molecules. To detect small glucose molecules, the ligand conjugates were used for a competitive assay. As the needle is inserted into a sample only containing the ligand conjugates (0 mg/dL of glucose), the conjugates were transferred across a membrane and bound to the immobilized con A, which results in a wavelength shift, as shown in Figure 15b. For the needle inserted into a sample containing 500 mg/dL of glucose, glucose molecules were transferred and bound to the con A by dissociating the conjugates, which reduces the wavelength shift. Figure 15c shows the different responses depending on the size of the membrane pores. The proposed device was able to detect the concentration range of glucose in human serum samples from 45 to 500 mg/dL, which is wider than the range of glucose levels in clinical use. The performance of the needle-type BLI sensor was compared with the standard BLI sensor, showing a better reproducibility with a comparable sensitivity. 

Table 1 below summarizes the recent progresses in FPI types, including their associated Q factors, refractive index detection limits, and target analyte with LOD, when available.

### 3.4. Tabulated Summary of FPI Sensors

Table 2 shows some examples of LODs for some of the target analytes shown in Table 1 with commercial biosensors for the comparison with FPI-based biosensors. 

## 4. Whispering Gallery Mode (WGM)-Based Biosensors

**Figure 16:**
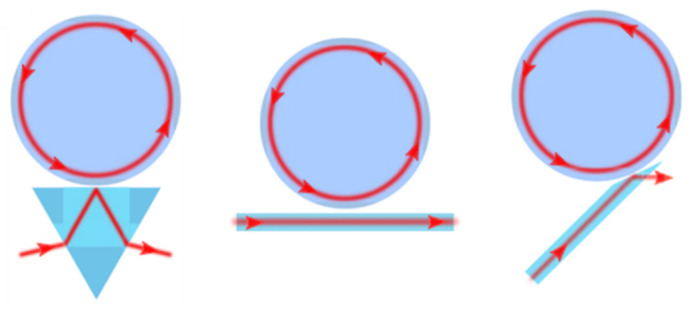
Coupling methods for WGM resonators. Reproduced from ref. [103] with permission from Elsevier.

Another increasingly pronounced class of high sensitivity optical resonators for use in biosensors is whispering gallery mode (WGM) resonators. Most works employing WGM resonators cite high Q-factors, high sensitivity, and rapid results, within micro- to nanoscale feature sizes. WGM-based biosensors are composed of a closed, radial cavity critically coupled with a prism, waveguide, or a fiber on its side or on the tip of a fiber as shown in Figure 16 [103,104,105,106,107,108,109]. WGM resonators confine light waves in any radial or polygonal enclosed surface, including rings, disks, spheres, cylinders, fibers, rods, or toroids, for example. They may also be categorized as either passive WGM or active WGM resonators, based on the lasing capability and coupling method. Active resonators make up the class that is capable of lasing, such as doped materials or resonators made of a gain medium. These are capable of being pumped remotely and collecting their spectra separately. Passive WGM must be directly coupled with a tunable laser source and detectors via fiber or waveguide [106,107]. These types do not generate light, but their resonance spectrum is tracked from the output transmission.

In a passive WGM resonator, the critical coupling condition is met when the light source’s resonant wavelength and linewidth matches that of the resonator, as indicated by a transmission minimum in its spectra. The resonant wavelength of the resonator, *λ_res_*, is given by

(8) $$\lambda_{res}\;=\;\frac{2\pi rn_{eff}}{m}$$

where *r* is the radius of the curvature, *m* is the mode number (integer value), and *n_eff_* is the effective refractive index, which changes with interactions on the resonator surface. The coupling (interaction) length (shown in Equation (9)) is proportional to the Q-factor of the resonator and its resonant wavelength.

(9) $$L\;=\;\frac{Q\lambda_{res}}{2\pi n}$$

where *L* is the coupling length, *Q* is the Q-factor, and *n* is the refractive index of the resonator material. Light waves travel the circumference via total internal reflection at the external barrier. Neglecting absorption or losses, the waves would continuously reflect along the boundary of the cavity. The surrounding material must be of a lower refractive index so that total internal reflection can occur. These travelling light waves eventually interfere constructively or destructively with newly arriving light waves from the waveguide. Physiochemical changes, such as those in bio-detection, modify the optical path length. The increased optical path length affects incoming light interactions with existing WGMs [17,24,104,107,110]. One or more detectors may be used to monitor spectral shifts, which are detectable when binding occurs on the surface of the resonator. The resonant wavelength shift due to such binding events can be expressed as

(10) $$\frac{\Delta \lambda }{\lambda }\;=\;\left[\frac{\alpha_{ex}\sigma }{\varepsilon_{0}\left(n_{r}^{2}\;-\;n_{b}^{2}\right)r}\right]$$

where *λ* and Δ*λ* are the resonant wavelength and resonant wavelength shifts, respectively, while α_ex_ is the excess polarizability of molecules and σ is the surface density due to binding activity. The permittivity in a vacuum is *ε*_0_, *r* is the radius of the curvature, and *n_r_* and *n_b_* are the resonator and buffer refractive indices, respectively [107].

**Figure 17:**
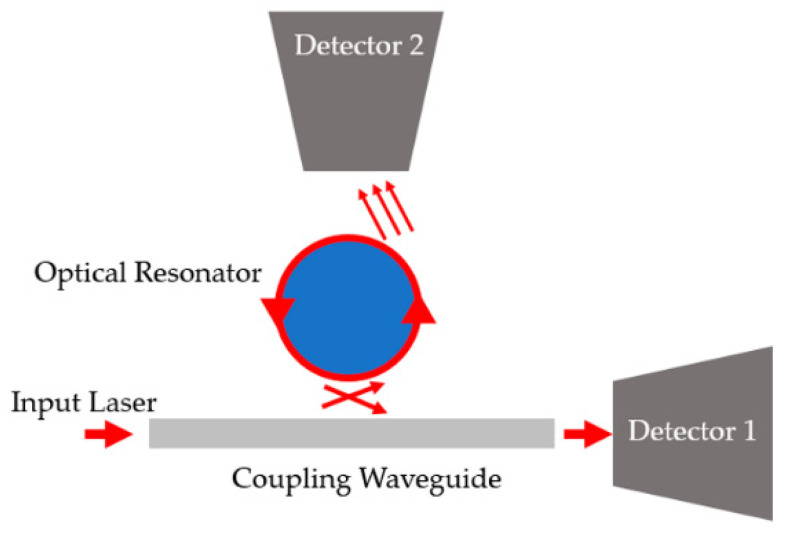
Example WGM resonator system. Reproduced from Open Access Article [107] in MDPI.

With respect to the system in which WGM resonators are equipped, advances such as transduction and data-processing or filtering techniques [21], dynamic referencing [111], surface functionalization [112,113,114], and the integration of microfluidics have been reported. Many WGMs are integrated into microfluidic chips to streamline testing with a small sample volume [104]. The resulting spectra is received through a detector typically in real-time. Figure 17 shows the system is typically constructed around a WGM resonator. For example, an input waveguide is coupled to the resonator, and the spectral output from both the input waveguide and resonator are recorded at separate detectors.

WGM biosensors are highly sensitive to changes in the surface conditions and refractive index, and they have been developed for single-molecule detection [20,21,115], pico- to atto-molar scale detection [21,112,113,116,117,118,119,120,121], and monitoring biological processes [122,123,124,125,126] or enzymatic reactions [127,128]. In recent studies, researchers have also taken advantage of geometric modifications [129,130] and material doping [116,118,119,131,132,133,134] to improve the Q-factors and sensitivity of WGM-based resonators. Geometric differences in most resonators may depend on fabrication capabilities or desired optical qualities, such as mode confinement, increased binding surface area, simpler fabrication techniques, or material choice. 

In the following sections, recently reported WGM resonator biosensors are organized by most to least common geometries. Novel material use, methods of fabrication, functionalization, and optical configurations are discussed, in addition to demonstrated biosensing capability, if any. A tabulated summary of the following is also provided in Table 3.

### 4.1. Ring- and Disk-Based WGM Biosensors

**Figure 18:**
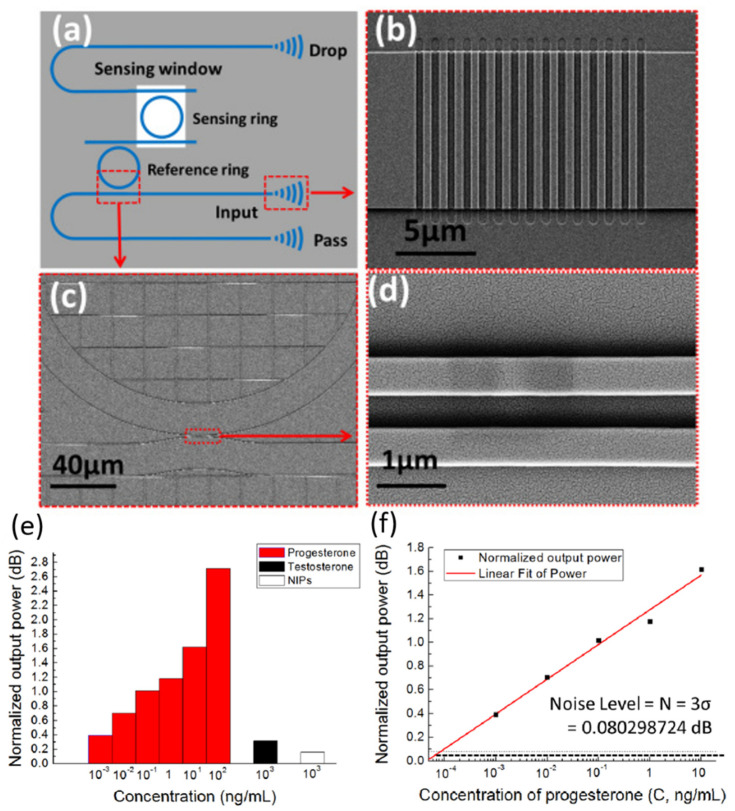
(**a**) Schematic of the CMRR setup. (**b**,**d**) SEM images of the grating used as couplers in different areas of the sensor, and (**c**) SEM of the reference ring coupled to the input waveguide. (**e**) Output power changes as a result of varying concentrations of progesterone (red), testosterone (black), and the NIP control (white), to demonstrate specificity. (**f**) Linear fit of power versus progesterone concentration. Reproduced from ref. [112] with permission © The Optical Society of America.

**Figure 19:**
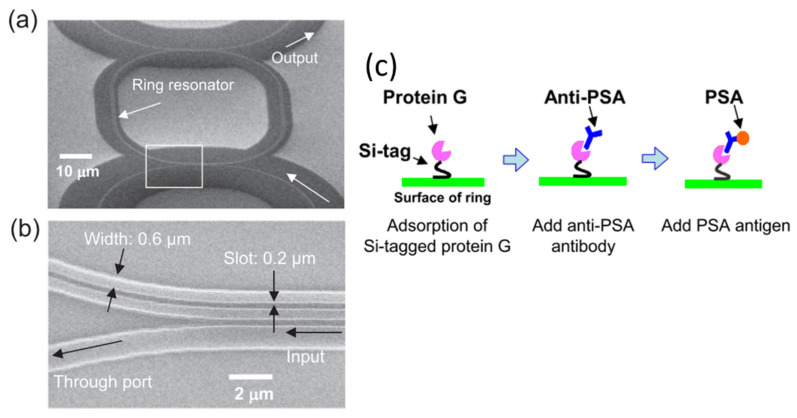
(**a**) SEM overview of the slotted ring coupled to the input and output waveguides. (**b**) a magnified image of the coupling point between the ring and the input waveguide. (**c**) Schematic of the procedure for PSA detection using protein G. Reproduced from ref. [114] with permission from Elsevier.

Disk and ring resonators have been investigated in a considerable number of recent studies. Figure 18c and Figure 19a below are sample SEM images of ring and disk geometries. 

Cascaded-micro ring resonators (CMRR) have been proposed to improve sensitivity of the ring resonator-based biosensors [112]. In a recent work by Xie et al., cascading was used to exploit the expansion of the FSR due to the Vernier effect, thus amplifying the measured signal changes. The CMRRs modified with molecularly imprinted polymers (MIPs) were used to detect progesterone. The waveguide and ring structures were first fabricated on SOI wafers via photolithography and etching processes, then the MIPs were formed using photopolymerization. The Q-factor for the reference ring was 1.7 × 10^3^, while that of the sensing ring was 1.0 × 10^3^, which is reduced further upon interaction. Non-imprinted polymer samples (NIPs) were exposed to the same post treatment and testing for the specificity test. The tunable laser light source is coupled to the CMRR chip’s input waveguide through a fiber array. Reference and output spectra are monitored using a power meter at the pass and drop ports, respectively, as shown in Figure 18a. An integrated microfluidic system was used to inject solutions into the chip. The normalized output power over time for the introduction of various progesterone concentrations was monitored in real-time, resulting in a linear relationship between log concentration of progesterone and the normalized output power as shown in Figure 18f. The average test time in these experiments was under 10 min with an LOD of 83.5 fg/mL. There was no significant power output change for Testosterone and NIP samples, which demonstrates the device’s specificity and the non-imprinted samples as a control as shown in Figure 18e.

Structural modifications that increase mode confinement and/or the available binding surface area were desirable [114,129,130]. For example, Taniguchi et al. implemented a slot waveguide structure into ring resonators for label-free detection of prostate-specific antigens (PSA) [114]. The silicon nitride (SiN) ring resonator, which has a superior thermal stability to a silicon ring resonator, is used as shown in Figure 19a,b. The slot width needed to be at least twice that of the experimental analyte to prevent steric hindrances, and its ideal dimension was determined by simulations. The ring was functionalized with protein G and the PSA antibody through silicon tagging (Si-tag) as the crosslink between Protein G and the SiN ring surface as depicted in Figure 19c. Protein G was preferred in this case due to its greater binding efficiency with the anti-PSA antibody. The resulting PSA detection limit was 10 ng/mL, which is near the clinical value (1 ng/mL).

**Figure 20:**
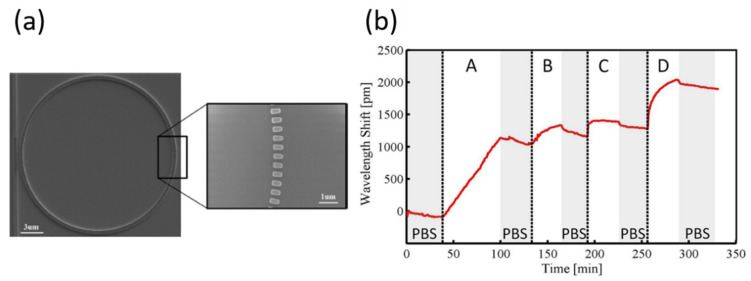
(**a**) SEM images of SWG ring resonator. (**b**) Wavelength shifts as a result of anti-streptavidin (A), BSA (B), streptavidin (C), and biotinylated BSA (D) as the target analyte. The shaded gray lines along the graph indicate PBS rinsing. Reproduced from ref. [129] with permission © The Optical Society of America.

Subwavelength gratings (SWG) shown in Figure 20a have also been reported to enhance the Q-factor and sensitivity of SOI ring resonators in biosensing [129]. Grating modification increased the evanescent field overlapping with attached analytes, thus increasing the sensitivity to binding events. The SWG ring resonators of various sizes and their respective circuitry were fabricated using electron beam lithography. The measured Q-factor of this system was 7000 and had a bulk sensitivity of 405 nm/RIU, for a probe wavelength of 1575 nm. The system’s best detection limit was 2.47 × 10^−4^ RIU using the resonator with a duty cycle (the ratio of the pattern width to the period of the grating) of 0.7. A sandwich assay of IgG, anti-streptavidin, streptavidin, and biotinylated BSA was used to demonstrate biosensing capabilities by monitoring wavelength shifts in real-time. As shown in Figure 20b, the total wavelength shift was nearly 2 nm, which is on a comparative order to most other works in this review.

**Figure 21:**
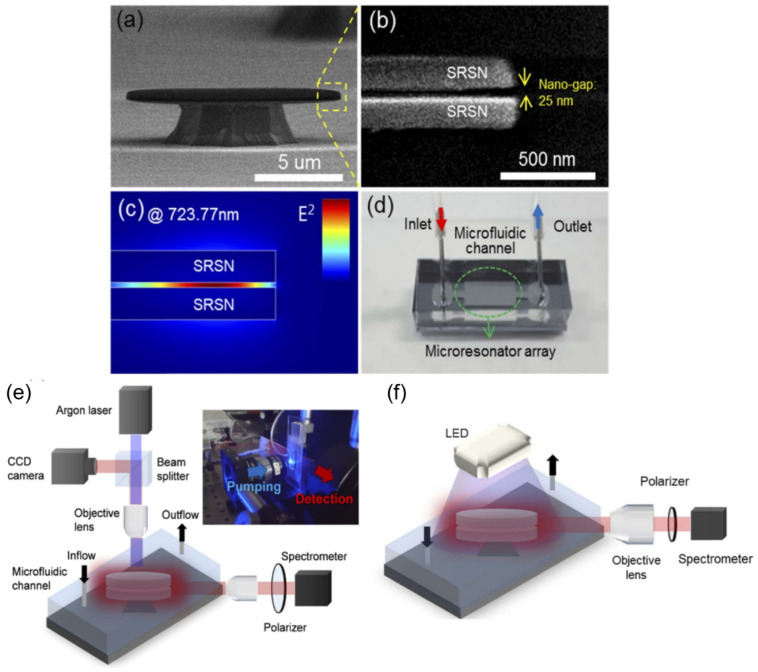
(**a**,**b**) SEM image of the microdisk resonator with a slot. (**c**) Intensity profile between the disks. (**d**) Microfluidic channel with the microdisk arrays. (**e**) Schematic of the proposed system. (**f**) Schematic of the system based on LED source. Reproduced from ref. [130] with permission © The Optical Society of America.

Another work introduced combining embedded silicon nanoclusters and a nano-gap in the on-chip resonator with a free-space optical pumping method [130]. Disks are formed on a substrate from silicon-rich silicon nitride (SRSN) film, and nanoclusters are formed from the SRSN film layers using an annealing process. Two disks at a time are arranged atop each other, with a 25 nm nano-gap between them, as shown in Figure 21a,b. Sensitivity enhancement is achieved through mode confinement in the nano-gap as presented in Figure 21c. The microdisk array is then formed inside the PDMS microfluidic channel as shown in Figure 21d. Demonstration of the sensor was done using a streptavidin-biotin complex at four different streptavidin concentrations. The chip is set under a focused argon (Ar) laser (457.9 nm), and resulting spectra are collected at a spectrometer arranged perpendicularly to the resonator as seen in Figure 21e. The Q-factor using the Ar laser was around 15,000 with a sensitivity of 0.012 nm/nM. Resonant shifts can be seen in real-time within a few minutes. The LED source was used in a simpler setup as shown in Figure 21f, but it was not used for the assay due to possible denaturation of proteins by UV. LED light pumping was reported to have a refractive sensitivity of 226.67 nm/RIU.

In another study, researchers achieved successful label-free, specific detection of *Staphylococcus Aureus* using a lysine K-functionalized WGM microdisk, giving way to an on-site, low cost, and rapid sensor for the virus [113]. Lysine K (Lys-K) is a capture antibody for *S. aureus*, and it was specific to the bacteria, in an E. coli-spiked dilution. Non-specific detection of bacteria has been demonstrated before, but the specific detection of bacteria in the presence of others is still currently being sought [135,136]. Silicon microdisks were first fabricated via photolithography followed by buffered oxide and dry etching steps. Afterwards, the surface was prepared for binding with hydroxylation, followed by PEG-Amine to attach Lys-K molecules. Transmission spectra were collected at a photodetector coupled with an oscilloscope. A probe wavelength of 630 nm was introduced to the microdisk using a tapered optical fiber. Over the course of 25 min, the maximum wavelength shift for *S. aureus* was 0.22 nm. As anticipated, no shift occurred in E. coli detection. Subsequent testing for four different concentrations of *S. aureus* were performed to determine the LOD. Based on those experiments and estimated surface density, the system was able to detect a minimum of 20 bacteria from a solution of 5 × 10^6^ CFU/mL. This translates to an LOD of 5 pg/mL. 

**Figure 22:**
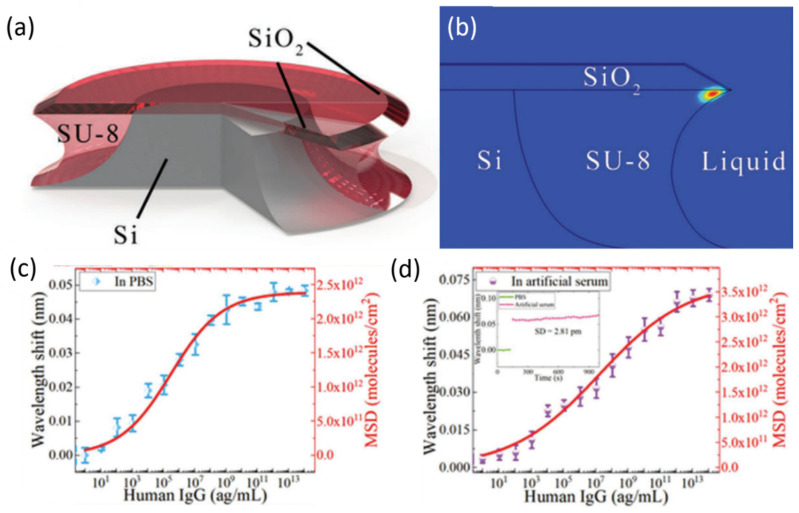
(**a**) HD structure. (**b**) mode field location based on simulations. (**c**) Shift in spectrum with human IgG in PBS. (**d**) Shift in spectra for human IgG in artificial serum. Reproduced from ref. [116] with permission from John Wiley and Sons.

Active WGM was demonstrated in a rhodamine-B doped silica microdisk in [116]. One advantage offered by active WGM devices over passive is natural excitation. Active types exploit inherent lasing properties, allowing remote excitation and collection of transmission. The sensor in this work successfully detected BSA and human IgG, in PBS and artificial serum. This is the first work integrating a silica microdisk on a hyperboloid drum (HD) trunk composed of silicon and dye-doped SU8 as the gain medium. Figure 22a presents the overall structure of the HD, and Figure 22b highlights the simulated mode field location. In their simulations, it was found that a certain wedge angle allowed for greater light–matter interaction where the lasing occurred. The predicted Q-factor of this resonator was 10^5^. For experiments, the whole HD-microdisk surface is functionalized, but the WGMs are confined in the inner drum where the doped photoresist was. Bulk refractive index experiments were performed and produced a sensitivity of 18.2 nm/RIU. GOPTS (3-glycidoxypropyltrimethoxysilan) was used to silanize the microdisks in preparation for BSA and IgG testing. The corresponding wavelength interrogation results for IgG in both PBS and artificial serum are shown below (Figure 22c,d). The detection limits of BSA in PBS and human IgG in both solutions were 4.5 ag/mL and 9 ag/mL, respectively. Compared with other references for their work, the HD microdisk in this study ranks among the lowest detection limits.

**Figure 23:**
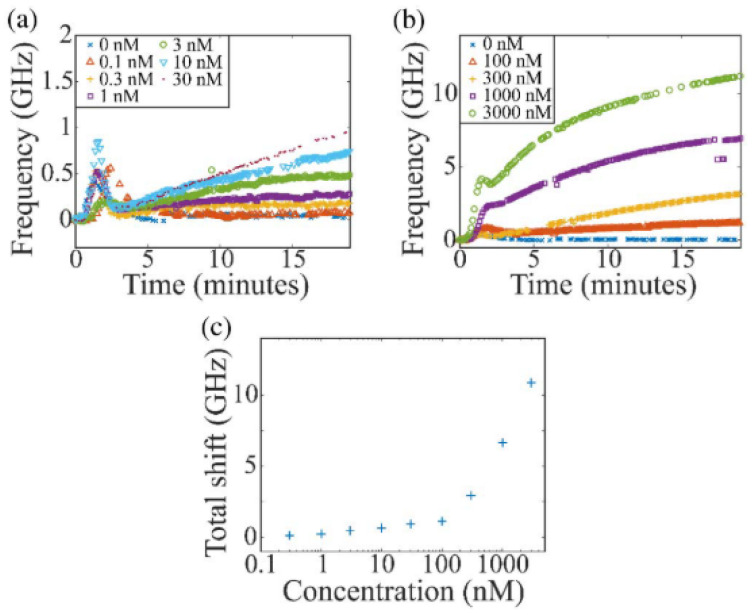
Frequency shifts as a result of protein rhS100A4 binding. (**a**) depicts the low set of concentrations ranging from 0–30 nM, while (**b**) depicts higher order concentrations up to 3000 nM. (**c**) is the calibration curve for log concentrations versus the total frequency shift. Reproduced from ref. [117] with permission © The Optical Society of America.

Another such work doped an aluminum oxide microdisk laser with Ytterbium ions (Yb^3+^) for active WGM sensing [117]. To fabricate the disks, radio frequency (RF) reactive cosputtering was used to form the Al_2_O_3_:Yb^3+^ film on silicon. Lithography and a reactive ion etching process were then used to form the desired geometry. They were then integrated onto PDMS chips containing microfluidic channels. Bulk refractive index and biodetection tests were performed after being placed in their optical setup. The quality factor of this device was 1.2 × 10^5^, and a sensitivity of 20.1 nm/RIU and LOD of 3.7 × 10^−6^ RIU was obtained from refractive index tests. An LOD of 300 pM was demonstrated for protein rhS100A4 (a tumor biomarker) in urine as shown in Figure 23. One major source of noise discussed in this work was its temperature sensitivity. This temperature sensitivity is less than that of silicon microdisks and its own refractive index sensitivity, however. The refractive index sensitivity for the sensor was 5.74 THz/RIU (6.02 pm/K), while the temperature sensitivity was an order of magnitude less, at 1.72 GHz/K (20.1 nm/RIU).

### 4.2. Spherical WGM-Based Biosensors

Spherical resonators provide some of the highest Q-factors among other geometries. These are typically based on microbubbles, microspheres, or microdroplets. Some notable modifications were label-free surface functionalization [120,123,137,138], doping [118,119], and self-referencing [111,120].

#### 4.2.1. Solid Microbubble and Microsphere WGM-Based Biosensors

Lead ions were specifically detected using a microbubble and GR-5 DNAzyme functionalization [120]. Fu et al. utilized a self-referencing method proven to reduce noise in an earlier separate work by Zhang et al. [139]. The self-referencing technique is employed by tracking the transmission from a reference mode, then applying a polynomial fit to produce a repeating saw-tooth pattern that can be subtracted as the “noise” from the sensing mode. With this, the group was able to achieve an LOD for lead ions that is well under the environmentally safe threshold. The microbubble was formed by heating a capillary tube and applying compressed air to expand the center into a microbubble. The bubble’s walls were then etched using an acid to thin the walls and allow for the desired mode distribution. The inside of the microbubble was then functionalized using positively-harged poly-L-lysine solution (PLL) bonded to negatively charged GR-5 DNAzyme. Then substrate strands were bound to the DNAzyme strands for lead ion detection. The lead ions were shown to cleave to the substrate strands from the surface. For testing, the microbubble and capillary were connected to a syringe to introduce analytes. A tunable laser source supplied light through a tapered fiber coupled to the side of the tube. The wavelength shifts were monitored through the other side of the tapered fiber at a photodetector and data acquisition program. Lead ion solutions of concentrations ranging from 0.1 pM to 100 pM were introduced, which established the sensitivity and LOD for lead in water solutions. The sensitivity was 0.4726 pm/pM, and the LOD was 15 fM. The Q-factor was 4.67 × 10^4^. Then, to demonstrate specificity, competing ions (Na^+^, Ca2^+^, Zn^2+^, K^+^, Cu^2+^, Fe^3+^, Fe^2+^, and Mn^2+^) were introduced in equal concentrations to the lead ions, 1 µM. For lead, the wavelength shift exceeded 20 pm, while the rest of the ions did not exceed a 1.1 pm shift. Lastly, 100 pM lead ion spiked and non-spiked filtered river water was compared. A blueshift of over 1 pm occurred for the spiked river water, while no significant shift was detected for the non-spiked river water.

**Figure 24:**
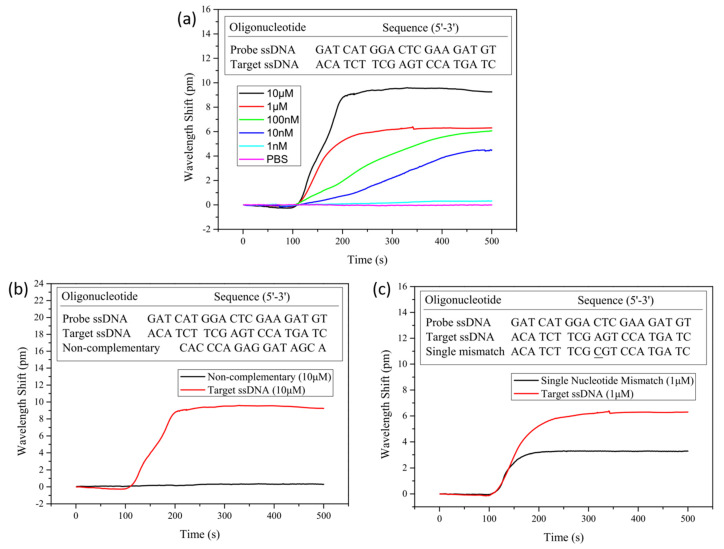
(**a**) Wavelength shifts from Target ssDNA binding over time. (**b**) Target ssDNA wavelength shifts compared to equal concentrations of non-complementary strands and (**c**) single nucleotide mismatch strands from. Reproduced from ref. [137] with permission from the author.

Real-time, label-free DNA detection using a PLL functionalized optofluidic microbubble WGM biosensor was demonstrated by Fu et al. in 2018 [137]. The system consisted of a tunable laser, filter and polarizers, tapered fiber coupled to microbubble, a photodetector, and a DAQ software on computer. For their label-free surface preparation following piranha cleaning, the probe ssDNAs were immobilized on the inner wall of the microbubble using PLL through electrostatic binding. Non-complementary, target, and mismatch ssDNAs in PBS buffer solution were introduced to detect the wavelength shift. The real-time shift in wavelength was monitored, and it took about 10 min per test. Results show that this device can differentiate between target, non-complementary and mismatch DNA with high selectivity. The LOD of the target ssDNA was 10 nM with a 4.46 pm wavelength shift (Figure 24a), while the shift for single-nucleotide mismatch and non-complementary strands were 3.28 and 0 pm, respectively (Figure 24b,c).

In another work, acrylate microspheres were used for label-free, real-time detection toward testing drugs/treatments using a non-invasive approach [123]. Cytochrome C (Cyt-C) is released by Jurkat cells in the body as a response to staurosporine, an anti-cancer drug. It is released upon cell apoptosis. A syringe pump is used to continuously flow the drug solution through a microfluidic chip containing the sample cells. Dying Jurkat cells release the analyte, which flows through a tube to the functionalized, acrylate polymer microbead. The microbead is positioned on a prism. Light from a 1310 nm laser is directed to the prism and bead setup, and the resulting reflected light is received at a photodiode. The microbead is treated by biotin-dextran so that a biotinylated antibody-streptavidin compound can be immobilized on the biotin while dextran passivates the surface to minimize nonspecific binding. An estimated LOD in complex media was 6.82 nM. To demonstrate real-time test capability, the Cyt-C emissions from treated, non-treated, and control cells were monitored over 5 h. There were significant changes in mass loading over 5 h for treated cells. A mass loading of over 600 pg/mm^2^ was recorded for drug-treated cells as a 300 pg/mm^2^ increase over the untreated group. The reception of Cyt-C and the increased mass loading from the WGM signal was confirmed by the decrease in cell viability in the MTT (3-(4,5-dimethylthiazol-2-yl)2,5-diphenyltetrazolium bromide) assays, which was used an alternate standard to verify the metabolic activity results gathered by the sensor.

#### 4.2.2. Liquid Crystal Microdroplet WGM-Based Biosensors

**Figure 25:**
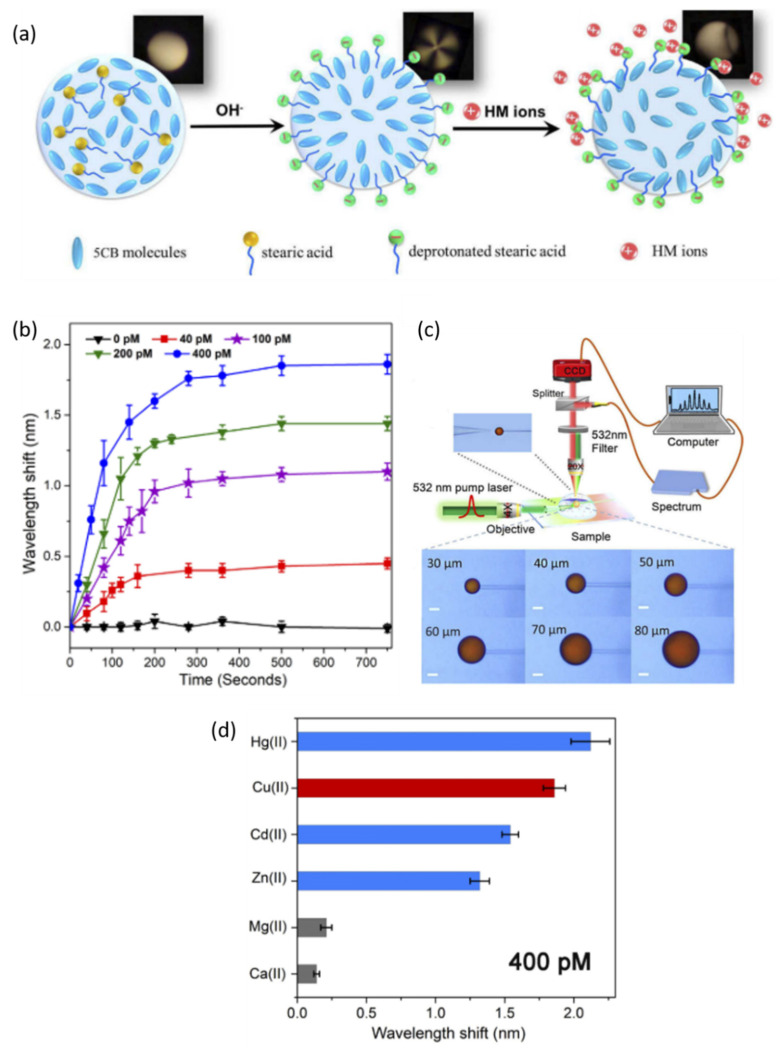
(**a**) Schematic of 5CB microdroplets detecting heavy metal ions. (**b**) Wavelength shifts associated with increasing concentrations of Cu(II) ions. (**c**) Schematic of the experimental setup used for HM ion detection. Droplets are fixed to the capillary connected to a syringe pump to control the droplet size in the medium. It is pumped by the laser shown through a tapered optical fiber. (**d**) specificity results for other heavy metals (blue), Cu(II), and acceptable minerals found in water (gray) at 400 pM concentrations. Reproduced from ref. [119] with permission © The Optical Society of America.

A liquid crystal (LC) microdroplet WGM resonator was used to detect heavy metal (HM) ions in real-time [119]. The LC 5CB microdroplet solution was composed of 1% 4-cyano-4′-pentylbiphenyl (5CB) in n-heptane, with equal doping of 0.01% stearic acid and fluorescent dye (DCM). As shown in Figure 25c, one microdroplet was suspended at a time from a tapered microtube in the host solution. Then DCM-doped microdroplets were pumped using a frequency-doubled pulse laser, through a tapered single mode fiber. WGM lasing spectra were collected using a spectrometer. Increased environmental pH was shown to change the anchoring state of the 5CB molecules at the LC/aqueous interface and increased the amount of deprotonated stearic acid. Deprotonated stearic acid consequently provides a receptor site for heavy metal ions, as demonstrated in Figure 25a. In this figure, the insets are images of the microdroplet taken using a polarized light microscope, illustrating the anchor state changes of LC as the pH increases without heavy metal ions and then repolarizes upon HM introduction. The WGM lasing spectra recorded for microdroplet sizes ranging from 30–80 µm for a constant pH, to optimize microdroplet size based on the desired transverse electric (TE) modes. As shown in Figure 25b, the experiments for the detection of copper (II) chloride were conducted for concentrations ranging from 0 to 400 pM using 60 µm droplets, resulting in an LOD of 40 pM, which is substantially less than the threshold set for water quality. Other heavy and light metal ions were also introduced at 400 pM to demonstrate a broad range of functionality as well as specificity to heavy metal contaminants. The most responsive in this system were mercury ions, with the least being magnesium and calcium (little to no toxicity) as presented in Figure 25d.

A year later in 2020 [138], the same research group demonstrated urea detection at an LOD of 0.1 mM with the same type of active LC microdroplet-based WGM biosensor using the stearic acid doping method. The same research group also reported urease detection using the LC microdroplet-based WGM resonator alongside a different functionalization method with 4-cyano-4′-biphenylcarboxylic acid (CBA) [128]. CBA was produced through UV exposure of the microdroplet, rather than adding a doping material. The resulting detection limit reported was 0.5 µg/mL. The sensor was specific to urease over other enzymes tested.

**Figure 26:**
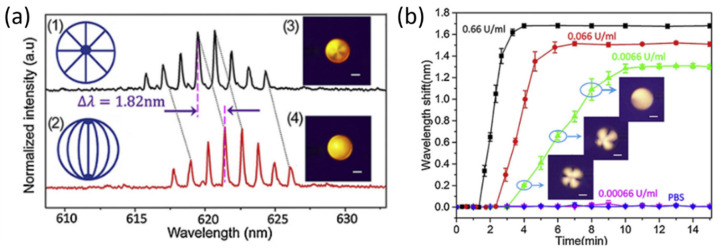
(**a**) Spectral shifts for corresponding configurations. (**b**) shift response for increasing concentrations of AChE. Reproduced from ref. [118] with permission from Elsevier.

In 2019, Duan et al. developed an active LC microdroplet-based WGM biosensor for real-time and label-free detection of acetylcholinesterase (AChE) and two pesticides (fenobucarb and dimethoate) [118]. The 5CB is employed again to enhance the WGM response, while fluorescent dye DCM is used for active WGM biosensing. Using an automated micropump, the droplets were formed in the medium. A fiber was used to pump light to the microdroplet, and the resulting emission was recorded with a spectrometer. The microdroplet is also visually monitored using a polarized light microscope. AChE hydrolyzes Myristoylcholine (Myr) on the microdroplet surface, which in turn changes the orientation of the liquid crystals and the dielectric constant. These changes result in the shift of the WGM lasing spectrum (Figure 26a), which then can be used to determine the concentration of AChE. As shown in Figure 26b, the detection limit for AChE was found to be 0.0066 U/mL from tests in a range of concentrations. Two pesticides, fenobucarb and dimethoate, were detected with this active LC microdroplet-based WGM biosensor using the inhibitory effects on AChE. In the presence of those pesticides, AChE’s function to hydrolyze Myr was limited, which was detected by the WGM lasing spectrum shift. The LODs for the fenobucarb and dimethoate are determined to be 0.1 pg/mL and 1 pg/mL, respectively.

### 4.3. Toroid WGM-Based Biosensors

Alternative to the preceding structures is the microtoroid, which have been shown to have the highest Q-factors of recent studies, on the orders of 10^5^–10^7^.

**Figure 27:**
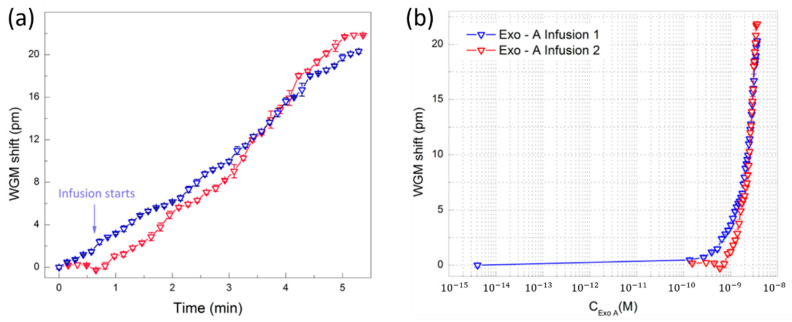
(**a**) Real-time responses of 2 different α-Exotoxin A conjugated microtoroid batches to Exotoxin A infusions in artificial sputum. (**b**) Wavelength shifts as a function of increased Exotoxin A concentration in artificial sputum. Adapted from ref. [140] with permission from ACS Publications.

Toren et al. recently accomplished the detection of Exotoxin A using a microtoroid WGM resonator [140]. Pseudomonas aeruginosa exotoxin A is a viral biomarker commonly indicative of cystic fibrosis (CF). The silicon microtoroids were formed using UV photolithography, followed by wet and dry etching processes. These were functionalized for biodetection using 3-(trihydroxysilyl) propyl methylphosphonate (THPMP) using EDC in MES, then anti-Exotoxin A was covalently bonded. A 1550-nm laser source was coupled to the microtoroid slide via tapered optical fiber and resonant wavelength shift was measured in real-time as shown in Figure 27a. Baseline and artificial Exotoxin-A spiked sputum samples resulted in spectral shifts of 8.49 pm and 20.04 pm, respectively, within 5 min. As shown in Figure 27b, the LOD for Exotoxin A using their unique surface functionalization method was 2.45 nM, with high specificity in diluted artificial sputum.

**Figure 28:**
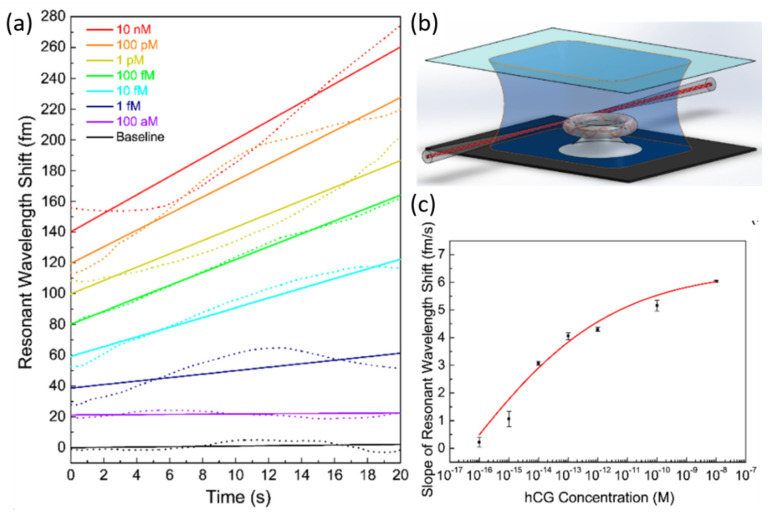
(**a**) Corresponding wavelength shifts over 20 s for concentrations of hCG, ranging from 100 aM to 10 nM. (**b**) Schematic of the microtoroid chamber and optical fiber. (**c**) concentration curve resulting from (**a**). Reproduced from ref. [121] with permission from ACS Publications.

Single-molecule detection was accomplished using a microrotoroid-based frequency-locked optical whispering evanescent resonator (FLOWER) with an improved signal-to-noise ratio [21]. This was accomplished using a balanced photodetector, a frequency laser-locking pre-filter, and post filtering in data acquisition. Solutions containing nanoparticles were introduced to the chip via a syringe pump. A step-finding algorithm was used for the accurate detection of single particle binding events. First, particle detection tests over a range of sizes (2.5 nm was smallest) were performed to characterize their system, then biosensing validation tests using exosomes, ribosomes, mouse IgG, and human interleukin-2 were conducted. The magnitudes of resonance shifts corresponded to the respective nanoparticle sizes and the number of binding occurrences. Silica nanoparticles were detected at a minimum concentration of 0.2 pM in water. FLOWER was later used for the label-free detection of human chorionic gonadotropin (hCG) in simulated urine samples [121]. These researchers also used post processing and electronic feedback control to improve their signal-to-noise ratio. Similar to their earlier work, the frequency of the resonator was tracked during testing and increased the data acquisition rate, rather than constantly scanning a laser wavelength. The disks were fabricated via wet then dry etching processes from silicon wafers with an oxide top layer. Only microtoroids found to have Q-factors of 10^7^ or greater were functionalized and used for testing. The toroids were treated with a silane linker, anti-hCG IgG antibody, and were then mounted to the measurement stage. Around this, a chamber was built to house sample fluids as depicted in Figure 28b. Solutions of hCG and simulated urine were introduced. Instead of repeated solution introduction, hCG was simply infused into the pre-existing solution. After 30 sec of each infusion, the resonant wavelength was recorded. The measured resonant wavelength shifts (dotted lines) and their corresponding linear fittings (solid lines) are depicted in Figure 28a. Figure 28c is the concentration curve developed from the aforementioned test, resulting in an LOD of 120 aM. Subsequently, real human urine samples diluted in the same simulated urine were tested. The lowest detectable hCG concentration with simulated and human samples were 1 fM and 3 fM, respectively. 

### 4.4. Capillary-Based WGM Biosensors

**Figure 29:**
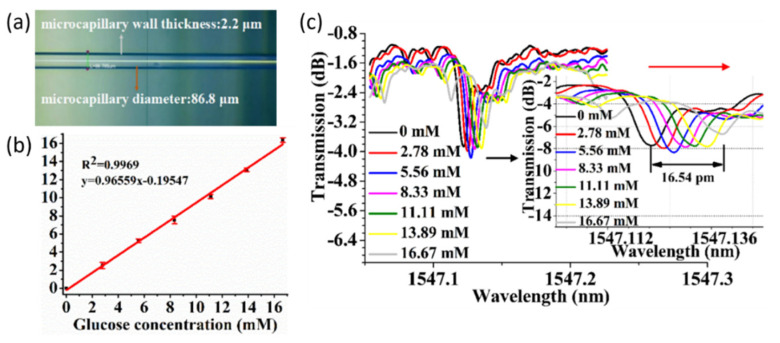
(**a**) Microscope image of the microcapillary resonator. (**b**) linear fitting for glucose concentration test performed in (**c**). (**c**) Measurement results for glucose detection. Reproduced from ref. [141] with permission © The Optical Society of America.

Capillary, or fiber-based WGM sensors have also been explored due to their ease of integration with microfluidics, large binding surface area, and high Q-factor [122,141]. Using a microcapillary (MC) fabricated from optical fiber, Wan et al. [141] were able to detect the concentration of glucose. The wall thickness of the capillary was optimized for sensitivity, based on the FDTD method for a capillary filled with a fluid. The MC was fabricated using an inflation and tapering method to achieve the dimensions depicted in Figure 29a. The capillary was excited using a tapered optical fiber attached to the tunable laser source. Spectra were recorded at a photodetector. The baseline sensitivity of 23.36 nm/RIU was established by introducing blank fluids of increasing refractive index. Once the capillary was functionalized using glucose oxidase and coupled to the fiber, fluids are introduced via a syringe pump. Transmission was monitored for a range of glucose concentrations from 2.78 to 16.67 mM as shown in Figure 29c. This produced a sensitivity of 0.966 pm/mM; the respective concentration curve is depicted in Figure 29b.

Using a biocompatible MC integrated with a microfluidic channel, Wang et al. were able to monitor conformational changes of G-quadruplex in real time [122]. The use of a MC to monitor DNA restructuring alleviates challenges associated with labeling and complex detection systems. G-quadruplex is currently being studied in other works for their role in cancer therapies. The folding of potential G-quadruplex forming sequences (PGQS), or formation of G-quadruplex, is involved in oncogene regulation [142]. After thinning the walls of a silica capillary with hydrofluoric acid, it was silanized, then PGQS was immobilized using streptavidin-biotin conjugate. The capillary was fixed to a glass slide and connected to a syringe pump by polyethylene tubing. Light from a tunable laser was coupled into the MC by a tapered optical fiber. Transmission was received at an optical power meter. A sensitivity of 995.63 nm/RIU was derived from simulation. K^+^ ions of increasing concentrations were introduced to induce the folding of PGQS into the stable G-quadruplex structure, and the resulting wavelength shifts were collected. As more ions were introduced, the slope of the relative wavelength shift over time decreased from −18.85 × 10^−5^ nm/s at 1 µM KCl to −2.03 × 10^−5^ nm/s at 10,000 µM KCl, due to reduced unfolded and single-stranded oligonucleotide availability as the experiment progressed. This work demonstrates the ability of a silica microcapillary WGM sensor as alternative candidate in the future for monitoring bodily responses to drug treatment.

Below in Table 3 is a summary of the WGM-based biosensors that were presented in this section. They are listed in order of appearance by geometry, material, Q factor, analytes, and their associated limit of detections.

**Table 3:** Summary of WGM-based biosensors.

Geometry	Material	Q	DL of RI	Analyte	Type of Sample	LOD	Ref.
Ring	Si	1.0 × 10^3^	9.7 × 10^−6^ RIU	Progesterone	DI water	83.5 fg/mL	[112]
Ring	SiN on SiO_2_	1.3 × 10^3^	141 nm/RIU	PSA	Tris-HCl	10 ng/mL	[114]
Ring	Si on SiO_2_	7.0 × 10^3^	2.47 × 10^−4^ RIU	Biotin-BSA	PBS	2.47 × 10^6^ RIU	[129]
Microdisk	SiN	1.5 × 10^4^	226.67 nm/RIU	Streptavidin	PBS	6.7 nM	[130]
Microdisk	Si	10^4^	-	*S. aureus*	TBS	5 pg/mL	[113]
Microdisk	SiO_2_	10^5^	18.2 nm/RIU	Human IgG	PBS/artificial serum	9 ag/mL	[116]
Microdisk	Al_2_O_3_:Yb^3+^	1.2 × 10^5^	20.1 nm/RIU	rhS100A4	DI water	300 pM	[117]
Microbubble	-	4.67 × 10^4^	265.2 nm/RIU	Pb^2+^	PBS	15 fm	[120]
Microbubble	-	-	1.216 nm/RIU	ssDNA	PBS	1 nM	[137]
Microbead	-	-	-	Cyt-C	PBS	6.82 nM	[123]
Microdroplet	Stearic acid-doped 5CB	10^3^–2.2 × 10^3^	-	Cu(II)	DI water	40 pM	[119]
Microdroplet	Stearic acid-doped 5CB	-	-	Urea	DI water	0.1 mM	[138]
Microdroplet	CBA	3000	-	Urease	DI water	0.5 µg/mL	[128]
Microdroplet	Dye-doped 5CB	3500	-	Fenubocarb, Dimethoate, AChE	PBS	0.1 pg/mL, 1 pg/mL, 0.0066 U/mL	[118]
Microtoroid	Si/SiO_2_	10^5^	-	Exotoxin A	Artificial sputum	2.45 nM	[140]
Microtoroid	-	10^5^–5 × 10^6^	-	SilicaNanoparticles	Water	0.2 pM	[21]
Microtoroid	SiO_2_	10^7^	-	hCG	PBS	120 aM	[121]
Capillary	-	1.3 × 10^6^	23.36 nm/RIU	Glucose	Sodium acetate	2.78 mM	[141]
Capillary	-	-	995.63 nm/RIU	G-Quadruplex conformation	PBS	-	[122]

### 4.5. Tabulated Summary of WGM-Based Biosensors

Table 4 shows some examples of LODs for some of the target analytes shown in Table 3 with commercial biosensors for the comparison with WGM-based biosensors. 

## 5. Conclusions and Outlook

Optical resonators have been considered as one of the most promising platforms to develop an ideal biosensor and have been actively investigated. In this review, we discussed recent progresses on the optical resonator-based biosensor development categorized into FPI-based biosensors and WGM-based biosensors. FPI-based biosensors are typically simpler and easier to fabricate than WGM-based biosensors, while WGM-based biosensors usually have a higher Q-factor and sensitivity. Very high Q-factor optical resonators have been successfully developed by modifying the configurations, materials, and light coupling methods, resulting in a very high sensitivity biosensing, even down to a single molecule detection. Label-free and real-time detection capabilities are also major advantages of any optical resonator-based biosensors. A wide range of sensing applications have been demonstrated with optical resonator-based biosensors, some of which show the capability of rapid and multiplexing detection. Efforts also have been made to integrate the optical resonator-based biosensors with microfluidic systems to reduce the required sample volume and assay time. Few portable optical resonator-based biosensors have been reported and there are efforts to reduce the overall cost of the system by employing low-cost components and simple fabrication processes. However, the challenges still remain for optical resonator-based biosensors to be developed further for the clinical use or for POC applications. The sensitivity and selectivity still need to be improved further to lower the LOD and enable the detection of smaller biomolecules. Demonstration of the device performance with crude clinical samples such as blood, serum, and urine instead of synthetically prepared samples in buffer solutions is a must. Only BLI-based biosensors out of various optical resonator-based biosensors have been successfully commercialized. To be commercialized, the stability and reproducibility of optical resonator-based biosensors must be demonstrated. For POC applications, the overall cost of the system has to be reduced, and it must be developed further into a portable and stand-alone system integrated with an automated microfluidic platform. A successfully developed optical resonator-based biosensor coping well with those challenges could greatly benefit medical diagnostics and accelerate the realization of an ideal POC device.

## Figures and Tables

**Table 1:** Summary of FPI-based biosensors.

FPI Type	Q	DL of RI	Target Analyte	Type of Sample	LOD	Ref.
FPI	-	10^−5^ RIU	-	KCl/NaCl solution	-	[37]
FPI	-	-	Biotin-BSA	DI water	2.82 pM	[41]
μFPI	128.4	593 nm/RIU	BSA	DI water	1 pM	[43]
Nanopore FPI	-	-	Aβ42, T-tau	Diluent/CSF	7.8 pg/mL, 15.6 pg/mL	[45]
Cascaded FPI	-	8.4 × 10^−7^ RIU (23,794.6 nm/RIU)	Streptomycin-BSA	PBS	1 ng/mL	[52]
FPI-DBR (SiO_2_/Ta_2_O_5_)	875	2 × 10^−9^ RIU	-	DI water	-	[36]
FPI-Surface Stress sensor	-	-	BSA	PBS	10 ng/mL	[40]
FPI with SCI	-	-	Anti-TG, Anti-TPO	Blood serum	1.7 IU/mL, 6 IU/mL	[53]
PSM	75	-	Bacteria	Double distilled water	20 bacteria/mL	[58]
PSM	59.8	-	19-base DNA	PBS	43.9 nM	[55]
PSM	-	5 × 10^−4^ RIU	8-base DNA	PBS	87 nM	[57]
PSM	-	-	Hydatid antigen	PBS	0.16 ng/mL	[61]
PSM	-	10^−3^ RIU (200 nm/RIU)	Atrazine pesticide	Water, humic acid	1.4 pg/mL, 0.8 pg/mL	[59]
PSM	-	10^−7^ RIU (1000 nm/RIU)	-	Water	-	[56]
PSM	-	350 nm/RIU	Streptavidin	DI water	500 nM	[60]
Fiber	-	7 × 10^−5^ RIU	Rabbit IgG	PBS	100 ng/mL	[69]
Fiber-Hydrogel	-	-	DTT	DI water	50 μM	[71]
Fiber-PDMS	-	-	Serum antibody	PBS	-	[72]
BLI (Blitz) *	-	-	Tau441 protein	MES/FBS	6.7 nM	[80]
BLI (Octet) *	-	-	GTX1/4 Aptamer, STX Aptamer	Water	50 pg/mL, 0.5 ng/mL	[91,92]
BLI (Octet) *	-	-	Testosterone	PBS	2.13 ng/mL	[93]
Needle-type BLI	-	-	Glucose	Human serum	45 mg/dL	[96]

* Commercial biosensors.

**Table 2:** Examples of LODs with commercial biosensors.

Type of Assay	Target Analyte	Type of Sample	LOD	Ref.
ELISA	Ab42	Plasma	0.3 pg/mL	[97]
Luminex xMap	Ab42, T-tau	CSF	60 pg/mL, 25 pg/mL	[98]
ELISA	Streptomycin-BSA	Swine urine	1.9 ng/mL	[99]
ELISA	BSA	PBS	0.38 ng/mL	[100]
ELISA	Anti-TG, Anti-TPO	Blood serum	25 IU/mL, 8 IU/mL	[53]
SPR	Atrazine	Water	20 pg/mL	[101]
ELISA	Rabbit IgG	Serum	3.4 ng/mL	[102]

**Table 4:** Examples of commercial biosensors.

Type of Assay	Target Analyte	Type of Sample	LOD	Ref.
ELISA	Progesterone	Milk	0.089 ng/mL	[143]
ELISA	PSA	Human serum	0.083 ng/mL	[144]
SPR	PSA	Serum	1 ng/mL	[145]
ELISA	Human IgG	Serum/plasma	1.6 ng/mL	[146]
ELISA	Cyt-C	Bloods	0.04 ng/mL	[147]

## Abbreviations

AChE	Acetylcholinesterase
APTES	(3-Aminopropyl)triethoxysilane
APTMS	(3-Aminopropyl)trimethoxysilane
BLI	Biolayer Interferometry
BPF	Band-Pass Filter
BSA	Bovine Serum Albumin
Con A	Concanavalin A
CMRR	Cascaded-Micro Ring Resonator
CSF	Cerebrospinal Fluid
Cyt-C	Cytochrome C
DBR	Distributed Bragg Reflector
DEP	Dielectrophoresis
DI	Deionized
DTT	Dithiothreitol
ELISA	Enzyme-Linked Immunosorbent Assays
FBS	Fetal Bovine Serum
FPI	Fabry-Perot Interferometer
FSR	Free Spectral Range
FWHM	Full Width at Half Maximum
GOPTS	(3-glycidoxypropyl)trimethoxysilan
GTX1/4	Gonyautoxin 1/4
hCG	human Chorionic Gonadotropin
HCPCF	Hollow-Core Photonic Crystal Fiber
HD	Hyperboloid Drum
HM	Heavy Metal
HS	Hyper-immune Serum
IgG	Immunoglobulin G
LC	Liquid Crystal
LOD	Limit-Of-Detection
MC	Microcapillary
MIP	Molecularly Imprinted Polymer
Myr	Myristoylcholine
PBS	Phosphate Buffer Saline
PDMS	Polydimethylsiloxane
PLL	poly-L-lysine
POC	Point-Of-Care
PS	Pre-immune Serum
PSA	Prostate-Specific Antigen
PSM	Porous Silicon Microcavity
Q-factor	Quality-factor
RIU	Refractive Index Unit
SEM	Scanning Electron Microscope
SPR	Surface Plasmon Resonance
SMF	Single Mode Fiber
STX	Saxitoxin
SWG	Subwavelength Grating
TBS	Tris-Buffered Saline
TE	Transverse Electric
THPMP	(3-trihydroxysilylpropyl)methylphosphonate
WGM	Whispering Gallery Mode
